# Vitamin D Supplementation During Pregnancy and Maternal and Neonatal Vitamin D Status at ≤32 Weeks Gestation: Romanian Prospective Observational Cohort Study

**DOI:** 10.3390/children12060682

**Published:** 2025-05-26

**Authors:** Ioana Andrada Radu, Manuela Cucerea, Cristian Gheonea, Radu Chicea, Dumitru Alin Teacoe, Bianca Ioana Mutică, Samuel Bogdan Todor, Gabriela Boța, Dragoș Popescu, Bianca Cosmina Coțovanu, Maria Livia Ognean

**Affiliations:** 1 Doctoral School, University of Medicine and Pharmacy, 200349 Craiova, Romania; ioanaraduandrada@gmail.com; 2 Faculty of Medicine, Lucian Blaga University, 550169 Sibiu, Romaniadumitrualin.teacoe@ulbsibiu.ro (D.A.T.); samuelbogdan.todor@ulbsibiu.ro (S.B.T.); maria.ognean@ulbsibiu.ro (M.L.O.); 3 Clinical County Emergency Hospital, 550245 Sibiu, Romania; 4 Department of Neonatology, George Emil Palade University of Medicine, Pharmacy, Science, and Technology of Târgu Mures, 540142 Târgu Mures, Romania; 5 Department of Pediatrics, University of Medicine and Pharmacy, 200349 Craiova, Romania

**Keywords:** vitamin D, 25(OH)D levels, pregnancy, vitamin D deficiency, vitamin D insufficiency, vitamin D supplementation, pregnancy outcome, preterm infant, prematurity

## Abstract

**Background**: Recently, vitamin D deficiency (VDD) has been described as a pandemic, affecting all groups of the population. Pregnant women and preterm infants are particularly vulnerable to vitamin D deficiency. **Objectives**: We aimed to evaluate the maternal and neonatal vitamin D status in relation with maternal vitamin D supplementations during pregnancy and to identify demographic, social, and healthcare risk factors for maternal VDD and vitamin D insufficiency in women delivering at ≤32 weeks of gestation. **Methods**: This prospective observational study was developed in the regional level III maternity unit of the Clinical County Emergency Hospital Sibiu. It included all admitted mothers who delivered at ≤32 weeks of gestation and their infants between 1 March 2022 and 28 February 2025. Infant deaths in the first 24 h of life, major congenital defects, chromosomal abnormalities, the admission of outborn infants without their mothers, or the transfer of the mother more than 48 h after birth were used as exclusion criteria. Maternal and neonatal data were collected from medical records. Data on maternal vitamin D supplementation were collected through interviews. Univariate and multivariate logistic regressions, linear regression, and predictive models were performed for data analysis. **Results**: A total of 146 mothers (median (IQR) age 30 (24–35) years) and their 164 preterm infants born at ≤32 weeks of gestation (median gestational age of 30 (27–31) weeks and birth weight of 1200 (900–1527) g) were included in this study. Only 43.15% of the mothers used multivitamins containing vitamin D during pregnancy, and 10.96% used specific vitamin D supplements. Vitamin D supplementation was used for a median of 4 (3–5) months at a median dose of 800 (250–1500) IU/day. Severe VDD (25(OH)D < 10 ng/mL), VDD (25(OH)D < 20 ng/mL), VDI (25(OH)D 20–29 ng/mL) were found in 19.86%, 55.48%, and 23.97% of the mothers and 16.46%, 58.53%, and 25.61% of their infants, respectively. A significant correlation was found between the maternal and neonatal status (r = 0.684, r^2^ = 0.468, *p* < 0.001, B = 0.62). Both the maternal and neonatal vitamin D status were correlated with the vitamin D duration and dose used for supplementation during pregnancy. The logistic regression analysis showed that birth during a cold season and increased parity are independently associated with severe maternal VDD, while birth during the cold season and a lower educational status were independently associated with maternal VDD. Only an absent vitamin D supplementation (in the form of a multivitamin or specific vitamin D supplements) has been proven as an independent risk factor for VDI. **Conclusions**: Our findings revealed a worrisome prevalence of severe VDD, VDD, and VDI in mothers delivering very prematurely and in their infants. Additionally, less than half of the mothers in this study used vitamin D supplements during pregnancy despite the national recommendations. The professionals involved in advising pregnant women and policymakers should find solutions to improve the vitamin D status in these vulnerable groups of the population.

## 1. Introduction

Over the past decade, inadequate levels of vitamin D—commonly referred to as the “sunshine vitamin” due to its essential role in supporting bone health—have emerged as a significant global public health concern, impacting individuals across all age groups. Numerous studies indicate that vitamin D deficiency (VDD) has become increasingly widespread worldwide [1,2,3,4,5,6,7]. According to the findings by Wimalawansa et al. [5], VDD is rapidly approaching pandemic levels, emerging as a significant health concern affecting various age groups and ethnicities. Recent research indicates that the prevalence of low vitamin D levels is increasing in both high-income and low-to-middle-income countries. Several factors contribute to insufficient vitamin D levels, including an inadequate dietary intake, a limited exposure to sunlight (including clothing and the use of sun-screen protections), geographic location, and higher latitudes [2,4,8]. Additionally, seasonal variations [4,8,9,10], skin pigmentation, ethnic backgrounds [8,10,11], and an increased body mass index (BMI) [9] significantly affect vitamin D statuses. The prevalence of vitamin D deficiency and insufficiency varies significantly worldwide, influenced by the cut-off values used for definition. Current estimates suggest that about 45% of the population may be affected [2]. The World Health Organization (WHO) has recently recognized vitamin D deficiency as a significant health concern, particularly concerning pregnancy outcomes and infant health. Consequently, the organization has updated its recommendations for vitamin D supplementation during pregnancy [12].

The research indicates that 55% to 83.28% of mothers of premature newborns have deficient vitamin D levels [9,13,14,15]. In Europe, the prevalence of inadequate vitamin D levels among mothers varies widely; recent studies demonstrate that the proportion of mothers with insufficient 25(OH)D levels ranges from 51% to 98.64% [3,9,14,16,17,18]. Similarly, studies are reporting an increased rate of VDD and vitamin D insufficiency (VDI) among preterm infants, with reported prevalence rates ranging from 60% [19] to 88.3% [10,14,18,20,21,22]. Severe VDD was reported in 49% of very preterm infants with a gestational age (GA) < 32 weeks [23] and in 74%of extremely preterm infants (GA 24–28 weeks) [24]. In Europe, Budic et al. [25] reported a cumulative VDD and VDI rate of 44% in preterm infants in Slovenia, while Zung et al. [14] reported a higher rate of 76% in Israel. Additionally, Matejek et al. [18] found the prevalence of VDD to be as high as 88.3% in the Czech Republic. The occurrence of 25(OH)D levels below 30 ng/mL, widely regarded by experts as indicative of VDI, was observed in 20% to 60% of the preterm infants [18,26]. Recent studies indicate a rising prevalence of vitamin D deficiency (VDD) and vitamin D insufficiency (VDI) among preterm infants, with reported rates ranging from 53% to 88.3% [10,14,19,20,21,22,26,27]. Significant disparities exist regarding the severe VDD, VDD, and VDI prevalence both globally and within Europe. These variations can be attributed to several factors, including the criteria used to define severe VDD, VDD, and VDI, the presence of national recommendations for vitamin D supplementation during pregnancy, variations in the dosages of vitamin D administered, the timing of evaluations for the maternal and neonatal vitamin D status, the methods employed to assess vitamin D levels, and the existence of national policies regarding the vitamin D fortification of food products.

The role of vitamin D in the intestinal absorption of calcium and phosphorus, as well as in the process of bone mineralization, has been well established over the years [28,29,30,31,32,33]. Recent research has highlighted additional effects of vitamin D, particularly its involvement in various aspects of development, including cell growth, differentiation, and lung maturation [23,34,35]. Moreover, vitamin D is essential for normal fetal brain development and plays a significant role in preventing brain injury [36,37,38]. It is also important in modulating the immune system and inflammatory responses [37,39,40]. Recent findings suggest possible associations between insufficient levels of vitamin D and the development of conditions such as bronchopulmonary dysplasia (BPD) [34,41], necrotizing enterocolitis (NEC), retinopathy of prematurity (ROP), neurodevelopment, and hyperbilirubinemia in premature infants [42]. Recently, multiple studies have analyzed and reviewed vitamin D’s actions as a neuroendocrine immune-regulating hormone, underscoring its significant implications in the nervous system [7,43,44].

Vitamin D metabolism undergoes important changes in pregnancy. Starting in the first trimester of pregnancy, there is a significant increase in the conversion of 25-hydroxyvitamin D (25(OH)D) to its active form, 1,25-dihydroxyvitamin D (1,25(OH)_2_D), which doubles during this period. This results in a three-fold elevation in the levels of active vitamin D. An additional increase in the vitamin D-binding protein enhances intestinal calcium absorption and facilitates the proper mineralization of the fetal skeleton [45,46,47]. The developing fetus must rely on the placenta for the 25(OH)D transfer from the maternal bloodstream as it cannot synthesize its own vitamin D [48]. Thus, the placenta plays a critical role in regulating the fetal vitamin D supply by actively taking up and metabolizing 25(OH)D [49,50]. A positive correlation of varying degrees, from moderate to strong, was found between the maternal vitamin D status near birth and the neonatal vitamin D status at birth [14,15,51,52,53,54]. Based on the existing evidence, Hollis et al. [55] have concluded that an inadequate maternal vitamin D status is the most important risk factor for VDD in preterm infants.

The research indicates that low levels of maternal vitamin D are associated with an increased risk of adverse pregnancy outcomes, including spontaneous abortions [56,57], preeclampsia [6,12,58,59,60,61], gestational diabetes [6,12,23,58,61], and premature birth [6,12,23,62,63,64,65]. In contrast, some studies have found no significant associations between lower maternal vitamin D levels or VDD and spontaneous abortions [9], preeclampsia [9,17,66], or preterm birth [16,66,67,68]. Additionally, insufficient vitamin D levels are linked to maternal hypertension [56,58,60], an increased likelihood of delivering a low-birth-weight (LBW) infant [6,56,60,62,63,67,69], or a small-for-gestational-age (SGA) newborn [6,12,57,60,62]. However, the evidence linking VDD and the delivery of an SGA infant and fetal birth weight remains controversial [22,64]. Higher levels of maternal vitamin D have also been correlated with a greater birth weight (BW) and head circumference [60,66,70], as well as improved fetal linear growth [7]. However, other researchers did not confirm these findings [22,64]. A recent meta-analysis by Liu et al. [7], which examined 42 studies published up to 2022, found that maternal supplementation during pregnancy with daily doses of vitamin D > 400 IU was associated with a decreased risk of intrauterine death, an increased maternal vitamin D status, and a reduced risk of VDI in the newborn compared to doses of <400 IU of vitamin D. However, the intervention did not affect the risk of preterm birth, LBW, neonatal BW, or head circumference. Other researchers [22,66] have also reported a beneficial effect of maternal vitamin D supplementation during pregnancy on the neonatal vitamin D status. Several factors may explain the varying and sometimes controversial findings in studies exploring the relationships between the maternal vitamin D status or supplementation during pregnancy and maternal, pregnancy, and neonatal outcomes. Possible explanations include inconsistencies in the definitions of VDD, VDI, and vitamin D sufficiency, differences in the timing of maternal vitamin D assessment or the initiation of vitamin D supplementation, and the use of different laboratory methods. Additionally, variations in dosages and formulations of vitamin D, as well as regional differences in lifestyle, nutrition, and national policies on vitamin D screening and supplementation for at-risk populations, particularly women of reproductive age and pregnant women, may further contribute to these discrepancies.

In Romania, research on vitamin D statuses remains limited. The most comprehensive study, conducted by Ene et al. [70] in 2018, involved 8594 participants aged 0 to 94 years from across the country, evaluating vitamin D levels between 2010 and 2017. This study revealed significantly higher 25(OH)D levels in women and children. Additionally, lower vitamin D levels were found during winter and spring, with the lowest values recorded in January. There were also regional differences in vitamin D statuses, with participants from Transylvania displaying the highest 25(OH)D levels. However, this study did not examine the vitamin D status of term and preterm infants or in pregnant women during pregnancy. Similar findings were reported by Niculescu et al. [71] in 2017 in the adult population for the southern part of Romania, by Bucurica et al. [72] in 2023 for adult patients hospitalized in Bucharest, and by Brîndușe et al. [73] in 2024. None of these studies included neonates or pregnant women. Although two other studies focused on children, neither included preterm infants, and no specific analysis was conducted on the newborns who were part of the studies [74,75]. Despite these gaps, the results of the Ene et al. [70] study prompted the Romanian Ministry of Health to develop a National Guideline for the Evaluation and Therapy of Vitamin D Status in Pregnant Women, Newborns, and Children [76] and a guideline for adults. Recently, Dragomir et al. [77] reported on the vitamin D status during pregnancy and in term neonates. Another report by Dragomir et al. [78] analyzed the vitamin D status during the first trimester of pregnancy and its relationship with gestational diabetes.

In the current epidemiological context in Romania, we aimed to analyze vitamin D supplementation during pregnancy and its association with the prevalence of VDD and VDI in women who deliver at or before 32 weeks of gestation. Additionally, we investigate the maternal vitamin D status and the influence of supplementation on pregnancy outcomes, as well as neonatal vitamin D levels. Recognizing the critical importance of vitamin D for maternal and neonatal well-being, this study seeks to identify actionable factors that can enhance vitamin D levels in these populations, who are particularly vulnerable to VDI and VDD.

## 2. Materials and Methods

### 2.1. Design

This study is a unicentric observational prospective cohort investigation conducted in the regional level III neonatal intensive care unit (NICU) at the Clinical County Emergency Hospital in Sibiu, Romania, from 1 March 2022 to 28 February 2025. Sibiu County is situated in the southern part of Transylvania, between the latitudes 45°28′ and 46°17′ north. The region features a moderate temperate–continental climate characterized by four distinct seasons and hot summers and cold winters. In the Sibiu area, sunlight availability fluctuates considerably throughout the year. From mid-April to October, the region experiences 5 to 8 h of sunshine daily. In contrast, the remaining months yield only 2 to 5 h of sunlight per day. The peak sun exposure occurs from June to August, while December and January record the lowest levels of sunshine [79,80].

### 2.2. Study Population

**Figure 1:**
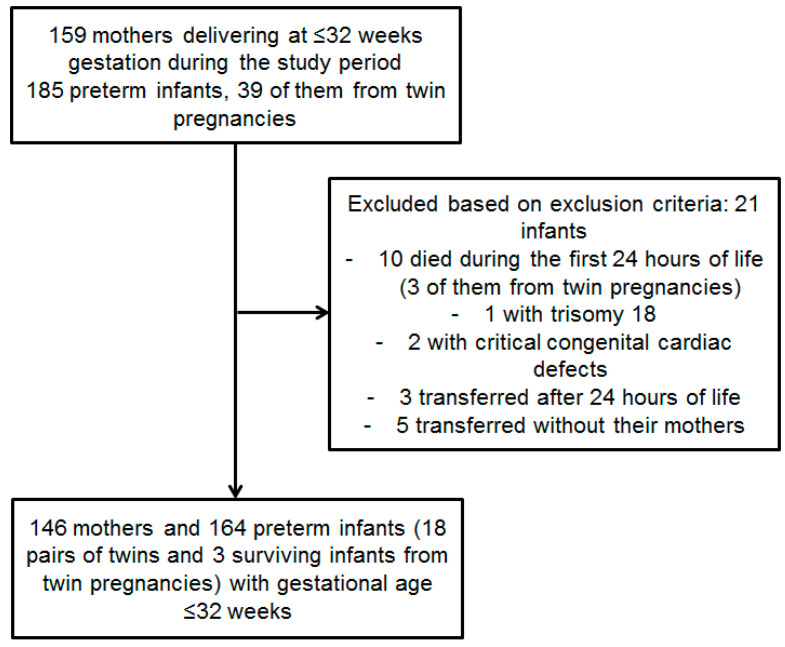
A flow chart of the study group selection.

This study included all women delivering at a gestational age (GA) of ≤32 weeks and their infants during the study period at the maternity ward of the Clinical County Emergency Hospital Sibiu, Romania. As a regional unit that follows the national mother and infant care policy, the neonatal intensive care unit (NICU) admits all preterm infants born at ≤32 weeks of gestation within the designated geographical area, along with their mothers. Infants were excluded from the study if they were admitted more than 24 h after birth, had major congenital anomalies or chromosomal abnormalities, or died within the first 48 h of life. The mothers of these excluded infants were not included in the analysis unless they could be paired with a surviving twin. Additionally, both the infant and the mother were excluded if the mother arrived at the NICU more than 48 h after delivery or was not admitted following the preterm infant’s transfer. Mothers who declined to participate in the study were also excluded. A flow chart detailing the selection of the study population is presented in Figure 1. This study complied with the ethical principles outlined in the Declaration of Helsinki. Informed consent was obtained from each mother for her and her infant’s participation in the study. This study was approved by the Ethics, Medical Deontology and Discipline Committee of the Clinical County Emergency Hospital Sibiu, as documented in the decision No. 22231/10.09.2021.

### 2.3. Data Collection

In this study, we gathered data on maternal characteristics, including age, number of gestations, parity, residence, educational attainment, and marital status, from maternal medical records. Our focus included various aspects of the pregnancy, such as pregnancy type and complications (including diabetes, hypertension, urinary tract infections, thyroid disorders, imminent preterm delivery, placental anomalies, and hemorrhages). We also examined the follow-up care provided by both the family physician and the obstetrician, as well as details about the rupture of membranes, delivery method, and any instances of fetal distress. The month of birth was also noted. Information about vitamin D supplementation—specifically, the types of supplements utilized and the duration of supplementation—was obtained through structured interviews following participants’ informed consent. Missing data from the medical charts were also collected through interviews. Additionally, we collected information on the GA, BW, Apgar scores, and gender of the preterm infants included in the study.

The preterm infants and their mothers were classified according to their vitamin D (VD) status. We categorized severe VD deficiency as serum 25(OH)D levels < 10 ng/mL, VD deficiency as levels < 20 ng/mL, an insufficient VD status as levels 20–29 ng/mL, and a sufficient VD status as levels ≥ 30 ng/mL, according to Holick et al. [28].

Neonatal serum 25(OH)D levels were assessed through a 10 µL blood sample collected within the first 12 h of life, coinciding with routine biochemical and hematological investigations. Maternal 25(OH)D levels were measured using the same method within 48 h post-delivery. This timeframe was selected to accommodate mothers who may have been transferred from lower-level healthcare facilities, as transfers typically occur 24 and 48 h after delivery if the infant is submitted to our unit. The analysis of 25(OH)D levels was conducted using the RapiRead Cube Reader (AFFIMEDIX, Inc., Hayward, CA, USA), which effectively detects and quantifies 25(OH)D_2_ and 25(OH)D_3_ collectively. With immunochromatography technology, the equipment can accurately detect 25(OH)D between 3 and 100 ng/mL using two highly specific monoclonal antibodies. The method has a variation coefficient of <1%, an accuracy of 98%, a specificity of 100%, a sensitivity of 3.3 ng/mL, and a correlation (r^2^) of 0.96 with the gold standard liquid chromatography with tandem mass spectrometry (LC-MS/MS) technology.

### 2.4. Statistical Analysis

Continuous variables were tested using the Kolmogorov–Smirnov test. Normally distributed continuous variables are reported as means and standard deviations (SDs), while the continuous variables with abnormal distributions are reported as the median and the first (Q1) and the third quartile (Q3). To assess correlations between variables, the Independent Samples T Test was employed for categorical variables. The Mann–Whitney U Test was used to locate differences between two independent groups regarding normally distributed variables, either ordinal or continuous. Risk ratios (RRs) were reported where appropriate. Additionally, a linear regression analysis was employed to evaluate the suggested correlations in univariate analyses. Binary logistic regression analyses using the Backward method were performed step by step to identify independent risk factors associated with vitamin D deficiency and insufficiency. To verify the accuracy of the identified multivariable model, we constructed a receiver operating characteristic (ROC) curve and evaluated the predicted probabilities for vitamin D deficiency. All statistical analyses were carried out using IBM SPSS Statistics for Windows, Version 23.0. A *p*-value of less than 0.05 was considered statistically significant, corresponding to a 95% confidence interval (CI).

## 3. Results

### 3.1. Participant Attributes

During the study period, 159 pregnant women either delivered or were admitted within 48 h after delivery at lower-level hospitals following a very preterm birth. Among these mothers, 21 delivered twins. We excluded 16 infants from the analysis due to early death, significant congenital abnormalities, or a late submission for preterm infants. Additionally, we excluded five mothers (and their infants) who were not transferred to our unit, as their vitamin D status was unavailable (Figure 1). The maternal and neonatal characteristics are detailed in Table 1. The median maternal age was 30 years (24–35 years). The preterm infants in the study group were born at a median GA of 30 weeks (27–31 weeks) and had a median BW of 1200 g (900.00–1527.50 g); the mean and range values are presented in Table 1. We noted that almost 65% of the mothers resided in rural areas, and approximately 40% were unmarried. Moreover, almost 15% were never seen during pregnancy by a family physician, and one-third of them had never consulted an obstetrician despite complications occurring in pregnancy in 68.9% of the cases. A delivery by cesarean section was necessary in 42.46% of the cases (Table 1).

### 3.2. Maternal and Neonatal Vitamin D Deficiency and Insufficiency

During pregnancy, 43.15% of participants (63/146) reported utilizing a vitamin D supplementation, while 10.96% (16/146) indicated the use of specific vitamin D supplements. The mothers reported a median (IQR) duration of supplementation of 4 (3–5) months. The daily median (IQR) dose was 800 IU (250–1500 IU). Based on the 25(OH)D analysis, we found that 19.86% (29/146) of the mothers had a severe VDD (25(OH)D levels < 10 ng/mL). Additionally, 55.48% (81/146) had a VDD (25(OH)D levels < 20 ng/mL), and 23.97% (35/146) had a VDI (defined by 25(OH)D values between 20 and 29 ng/mL). Notably, only 20.55% (30/146) of the mothers delivering very preterm infants had a sufficient vitamin D status at birth ((25(OH)D ≥ 30 ng/mL). The same trends were observed in the preterm infants: 16.46% (27/164) had a severe VDD, 58.53% (96/164) had a VDD, 25.61% (42/164) had a VDI, and only 15.85% (26/164) of infants had a sufficient vitamin D status at birth.

### 3.3. Correlations Between Maternal and Neonatal Vitamin D Status at Birth

The maternal and neonatal median (IQR) values of 25(OH)D were close to each other—18.20 ng/mL (11.975–26.675 ng/mL) and 18.55 ng/mL (11.925–28.100 ng/mL), respectively—similarly to the mean values, presented in Table 1. A significant correlation was observed between the maternal and neonatal vitamin D status (r = 0.684, r^2^ = 0.468, *p* <0.001, B = 0.62), as shown in Table 2. Moreover, statistically significant correlations were found between the duration and daily dosage of the vitamin D supplementation during pregnancy and the mothers’ and newborns’ vitamin D status (Table 2). However, no correlations were identified between the neonatal vitamin D status and the GA or BW (Table 2).

### 3.4. Maternal Vitamin D Status and Seasonality

**Figure 2:**
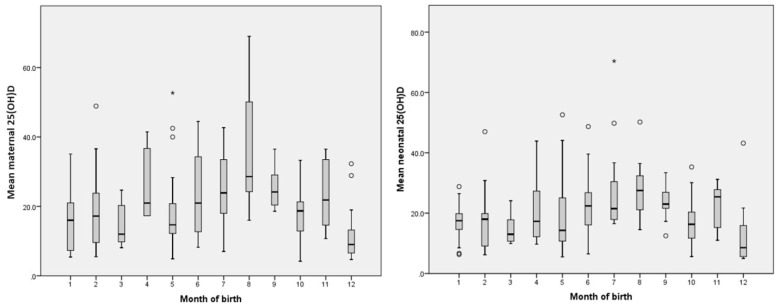
The distribution of the mean maternal and neonatal 25(OH)D values according to the month of birth.

The distribution of births across the seasons—winter, spring, summer, and autumn—was equal, with both the cold season (winter and spring) and the warm season (summer and autumn) accounting for 50% of the births each (Table 1). The analyses of the mean (SD) values for maternal and neonatal factors by the birth month showed a similar trend: lower values were recorded from December to March and again in May, with the lowest values occurring in December and March for both mothers and their infants (Figure 2). The mean (SD) levels of 25(OH)D began to rise in June, peaking in August, then declining in September, before increasing once more in November (Figure 2).

**Figure 3:**
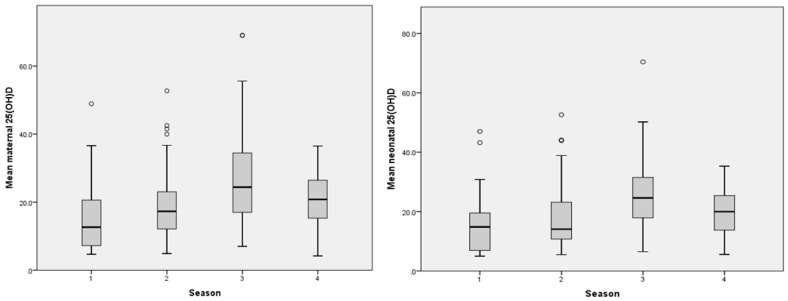
The distribution of the mean maternal and neonatal 25(OH)D values according to the season of birth. Legend: 1—winter, 2—spring, 3—summer, and 4—autumn.

The analysis of the maternal and neonatal vitamin D status by the season of birth—categorized as cold and warm—indicated that mean values were significantly lower during the cold season, with the lowest levels observed in winter. In contrast, higher mean values were recorded in the warm season, reaching their peak during summer (Figure 3).

### 3.5. Vitamin D Deficiency and Insufficiency and Association with Maternal Characteristics and Pregnancy Outcomes

The mothers in this study were categorized based on their vitamin D status. Severe VDD was defined as a 25-hydroxyvitamin D (25(OH)D) level of <10 ng/mL, while deficiency was characterized by levels < 20 ng/mL, and insufficiency was indicated by levels < 30 ng/mL. To identify factors associated with severe VDD, mothers with 25(OH)D levels < 10 ng/mL were compared to those with levels ≥ 10 ng/mL (see Table 3). Several factors were found to significantly increase the risk of severe VDD: a higher number of gestations and births, lower educational attainment, giving birth during the cold season, and being unmarried. Conversely, taking any multivitamin containing vitamin D or using vitamin D supplements was associated with a decreased risk.

When comparing mothers with 25(OH)D levels < 20 ng/mL to those with levels ≥ 20 ng/mL, several factors were significantly associated with severe VDD. These factors included an increased parity, lower educational status, single parenthood, births during the cold season, and a lack of obstetrical care during the pregnancy (Table 3). Importantly, maternal vitamin D supplementation—whether through multivitamins or standalone specific vitamin D supplements—along with higher vitamin D doses were linked to a reduced VDD risk.

Furthermore, when comparing mothers with 25(OH)D levels of 20–29 ng/mL (VDI) to those with levels ≥ 30 ng/mL, several demographic and health-related factors were identified as being linked to an increased likelihood of VDI. These factors include an increased parity, lower educational level, single parent status, and lack of obstetrical evaluations during pregnancy (Table 3). Longer durations of vitamin D supplementation—using either multivitamins or specific vitamin D supplements—during pregnancy and increased daily doses of vitamin D were identified as potential protective factors against an insufficient vitamin D status (Table 3).

### 3.6. Vitamin D Deficiency and Insufficiency and Association with Neonatal Characteristics

We examined the relationship between the maternal vitamin D status—categorized as severe deficiency, deficiency, and insufficiency—and the GA, BW, Apgar scores at 1 and 5 min, and neonatal vitamin D status. The comparison of infants born to mothers with a severe VDD and those born to mothers with a 25(OH)D level ≥ 10 ng/mL revealed significant differences in neonatal 25(OH)D levels, the prevalence of severe neonatal VDD, and the overall neonatal vitamin D status (Table 4). Similar associations were observed for mothers with VDD and VDI. However, we found no correlation between the maternal vitamin D status—whether categorized as severe deficiency, deficiency, or insufficiency—and neonatal outcomes such as the GA, BW, gender, or Apgar scores at 1 and 5 min (Table 4).

### 3.7. Risk Factors for Vitamin D Severe Deficiency, Deficiency, and Insufficiency

**Figure 4:**
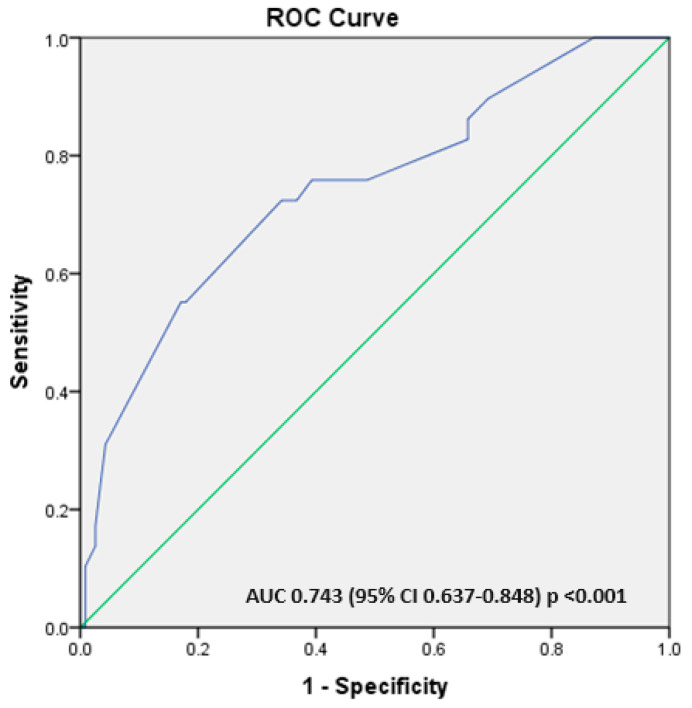
The predictive model using the independent risk factors for a severe maternal vitamin D deficit at birth using the variables identified as independent risk factors for severe vitamin D deficits (delivery during cold season, prolonged preterm rupture of membranes > 18 h, and increased parity). Legend: AUC—area under the curve and ROC—receiver operating characteristic.

We used binary logistic regressions, the Backward method, to identify the factors independently associated with the maternal vitamin D status during pregnancy. All maternal variables with a *p* < 0.200 were introduced in the analysis and eliminated step by step if not significant. Thus, an increased parity and birth during the cold season were the only independent factors associated with severe maternal VDD during pregnancy (Table 5). A receiver operating characteristics (ROCs) analysis was used to evaluate a predictive model built incorporating parity and deliveries during the cold season. The model has a good accuracy for predicting severe VDD as indicated by the area under the curve (AUC), with a value of 0.742 (95% CI 0.637–0.848) and *p* < 0.001 (Figure 4).

**Figure 5:**
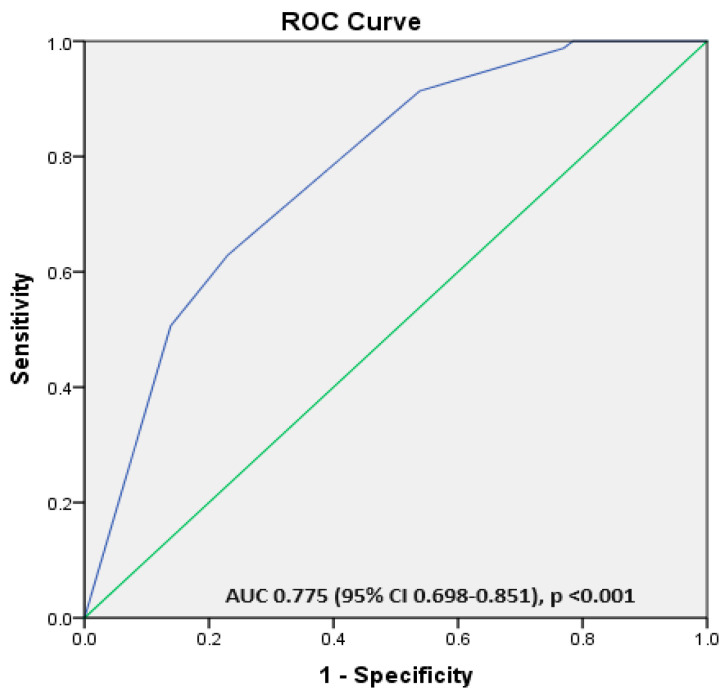
The predictive model using the independent risk factors for a severe maternal vitamin D deficit at birth using the variables identified as independent risk factors for severe vitamin D deficits (low educational level, delivery during cold season, and absent supplementation during pregnancy using vitamin D supplements). Legend: AUC—area under the curve and ROC—receiver operating characteristic.

The analysis was repeated to find the independent factors associated with maternal VDD during pregnancy. The findings indicate that giving birth during the colder months significantly increases the risk of maternal VDD. In contrast, a higher educational attainment and specific vitamin D supplements during pregnancy were found to reduce this risk significantly (Table 6). Furthermore, the AUC of the predictive model, which was developed based on these variables and evaluated using the ROC analysis, demonstrated a high accuracy in predicting maternal vitamin D deficiency, yielding an AUC of 0.775 (95% CI: 0.698–0.851), *p* < 0.001 (Figure 5).

**Figure 6:**
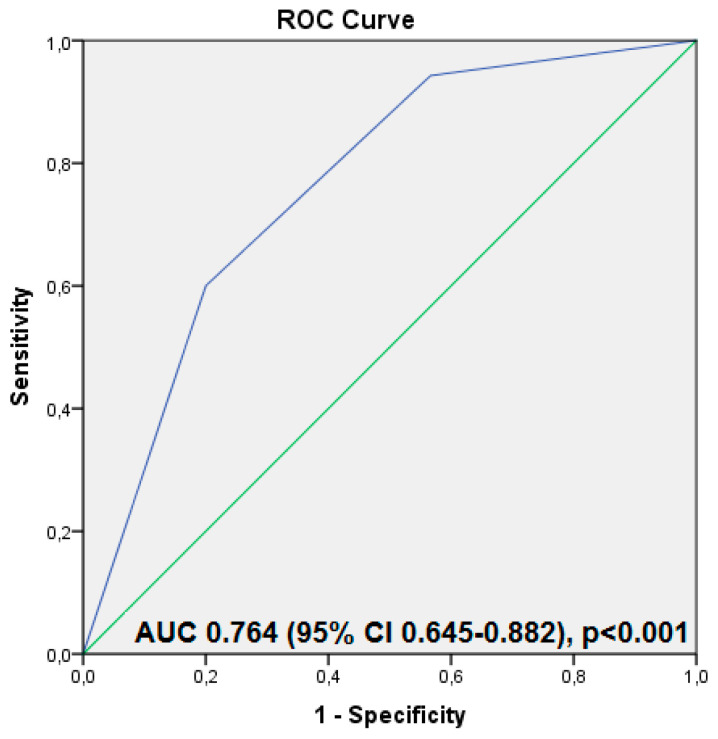
The predictive model using the independent risk factors for a severe maternal vitamin D deficit at birth using the variables identified as independent risk factors for severe vitamin D deficits (supplementation during pregnancy using multivitamin containing vitamin D and vitamin D supplements). Legend: AUC—area under the curve and ROC—receiver operating characteristic.

The binary logistic regression analysis has shown that using multivitamin supplements containing vitamin D to meet vitamin D requirements during pregnancy is ineffective and is linked to an increased risk of VDI. Conversely, a supplementation with specific vitamin D products is independently associated with a reduced risk of VDI (Table 7). The predictive model, based on the predictive probabilities, revealed an AUC of 0.794 (95% CI: 0.694–0.894), *p* < 0.001, which is highly predictive of maternal VDI (Figure 6).

## 4. Discussion

This study represents the first comprehensive analysis in Romania regarding the effects of maternal vitamin D supplementation during pregnancy on the vitamin D status of both mothers and neonates and on pregnancy outcomes for those delivering prematurely at ≤32 weeks of gestation. Our findings indicate a high prevalence of VDD, with rates of 55.48% observed in mothers and 58.53% in their infants. Additionally, the incidence of severe VDD, defined as 25(OH)D levels < 10 ng/mL, is concerning, affecting 19.86% of mothers and 16.46% of infants. A significant positive correlation was identified between the vitamin D status of mothers and neonates and the utilization of a vitamin D supplementation during pregnancy. Notably, longer durations and higher doses of vitamin D-specific supplements were associated with an improved vitamin D status in both mothers and infants, highlighting the importance of these interventions for enhancing health outcomes.

Recent research has significantly advanced our understanding of vitamin D’s mechanisms of action and metabolites, highlighting various effects that extend beyond its established role in supporting bone health. This suggests that the benefits of achieving and maintaining optimal vitamin D levels may encompass a broader range of health outcomes [2,5,23,33,35,56,81]. However, despite the growing interest in this area, a consensus remains elusive regarding the appropriate cut-off values for defining VDD or VDI. Furthermore, it is uncertain whether routine screening protocols or individualized assessments of the vitamin D status should be implemented in high-risk populations to formulate effective supplementation strategies aimed at achieving adequate vitamin D levels.

### 4.1. Particularities of the Study Group

The median (IQR) maternal age of the 146 mothers included in this study was 30 (24–35 years). Almost 65% of the mothers lived in rural areas, 40% were unmarried, and two-thirds had low to medium educational levels. Approximately 15% of the mothers did not have check-ups with a family physician, and one-third did not receive specialized obstetrical care during their pregnancy, despite the state’s guarantees for maternal and child healthcare. These factors not only indicate a higher risk of inadequate care during pregnancy but also increase the likelihood of complications. The assessment of maternal dietary habits found that only one mother adhered to a restrictive diet (Table 1). Various complications during pregnancy were noted in 68.9% of the cases. A significant association between VDD and complications during pregnancy and labor was recently reported by Alanazi et al. [82] (*p* <0.001). Less than half of the mothers surveyed reported taking vitamin D supplements during pregnancy. The majority opted for multivitamins that included vitamin D (47 out of 146), albeit for a limited duration (median of 4 months). Additionally, nearly all participants indicated that they initiated the supplementation in the second trimester of pregnancy. Furthermore, a lack of vitamin D supplementation during pregnancy, shorter durations, and lower dosages of vitamin D were associated with reduced vitamin D levels in both mothers and their newborns.

The preterm infants in the study group were born at a median (IQR) GA of 30 (27–31) weeks of gestation and with a median (IQR) BW of 1200 (900–1527.5) g, most of them presenting a relatively good condition at birth as suggested by the Apgar scores at 1 and 5 min (Table 1).

### 4.2. Maternal and Neonatal Vitamin D Status at Birth

Lower values of 25(OH)D during pregnancy have been linked to an increased risk of adverse pregnancy and birth outcomes, as evidenced by the findings of several studies [58,61,62,63,83]. Thus, lower maternal 25(OH)D levels were associated with an increased risk for various complications, including respiratory distress syndrome (RDS) [35,84], retinopathy of prematurity [85], SGA [62], a lower bone mineral density in early infancy [86,87,88], and an increased risk of osteopenia and rickets [61]. Furthermore, they are associated with atopy and/or allergy [89], negative affectivity during infancy [90], and attention-deficit disorders and autistic traits [91]. Moreover, a lower maternal vitamin D status has been correlated with an increased risk for a lower neonatal vitamin D status [14,15,51,52,53], lower Apgar scores at 1 and 5 min [19,92], and respiratory complications, such as RDS and bronchopulmonary dysplasia (BPD) [19,20,23,24,27,35,52,68,84,92,93,94,95,96,97,98]. Other concerns include inflammation [99,100]; early- and late-onset neonatal sepsis [19,97,100,101]; metabolic bone disease [19,86]; adverse neurodevelopmental outcomes, such as reduced cognitive abilities or epilepsy [102,103], attention-deficit/hyperactivity disorders, and autistic traits [91]; retinopathy of prematurity [85,104]; longer hospitalization lengths [92,101]; and increased mortality [24]. Given the potential adverse effects of maternal and neonatal lower vitamin D statuses, we are concerned about the low 25(OH)D values observed in both our study groups—mothers and their preterm infants.

Recent findings by Tofe-Valera et al. [52] in Spain indicate values similar to ours for the maternal median 25(OH)D values in umbilical cord blood at birth. They reported levels of 18.6 ng/mL compared to 18.2 ng/mL observed in our study. This research involved 52 preterm infants born at or before 32 weeks of gestation. Notably, the mean 25(OH)D values identified in our study (20.96 ± 12.57 ng/mL) were higher than those reported in the Spanish study (17.97 ± 9.35 ng/mL). Furthermore, a study conducted by Matejek et al. [105] in the Czech Republic presented lower mean maternal and neonatal 25(OH)D levels in umbilical cord blood from 127 preterm infants with a BW <1500 g, with values recorded at 41.2 ± 25.2 nmol/L for mothers and 29.9 ± 17.8 nmol/L for infants. These discrepancies may be attributable to differences in the timing and/or blood sampling method used for the vitamin D status assessment [106].

Our study found a significant positive correlation between the duration and daily dose of vitamin D supplementations during pregnancy and the maternal and neonatal vitamin D status (Table 2) with other reports in the literature [51,55,66,88]. The maternal vitamin D status demonstrated a weak negative correlation with the GA at birth, similarly to the findings of Anderson-Berry [107]. No correlations were found between maternal 25(OH)D levels and the infant’s BW or between the preterm infant’s vitamin D status and their GA and BW (Table 2), which is consistent with the results of the univariate analysis (Table 4). A weak correlation between maternal 25(OH)D levels in the umbilical cord blood at birth and the GA and BW was reported by Liu et al. [94]. A significant correlation between the neonatal vitamin D status and GA and BW (*p* < 0.05) was reported by Ardastani et al. [20]. However, the preterm infants in this study had GAs ranging between 28 and 37 weeks. Consistent with other studies [15,18,51,52,53], we found that neonatal 25(OH)D levels were positively and significantly associated with maternal levels, with a correlation coefficient of r = 0.684, r^2^ = 0.468, *p* < 0.001, and B = 0.62. This indicates that a 1 ng/mL increase in maternal 25(OH)D levels is associated with a corresponding increase of 0.62 ng/mL in neonatal 25(OH)D levels. Zung et al. [14] reported an even stronger association between the maternal and neonatal vitamin D status (r = 0.850, *p* < 0.001). However, the preterm infants in their study had higher GAs and BWs than ours. Maternal blood flow is the sole source of vitamin D for the developing fetus. Recent studies revealed that a placental endocytic mechanism, similar to that in the kidneys, is responsible for the uptake of vitamin D and its binding to vitamin D-binding protein and albumin [50,108]. This internalization of vitamin D occurs through a megalin–cubilin mediated process, which is essential for maintaining vitamin D homeostasis not only in the kidneys and parathyroid glands but also in the placental decidual cells and syncytiotrophoblast [50,108,109,110]. These findings underscore the reliance of the fetus and preterm neonate on the maternal vitamin D status.

In the univariate analysis, severe neonatal VDD was associated with severe maternal VDD (*p* < 0.001), and neonatal VDD was significantly associated with maternal VDD (*p* < 0.001). Additionally, neonatal VDI was correlated significantly with maternal VDI (*p* = 0.002). Notably, we also found a significant association between severe maternal VDD and VDD with severe neonatal vitamin D deficiency (*p* < 0.001 for both severe VDD and VDD) (Table 4).

### 4.3. Seasonality and Its Influence on Maternal and Neonatal Vitamin D Status

Romania has a temperate continental climate, with cold winters and hot summers, and moderate sun exposure during spring and autumn [73]. Situated at latitudes between 45°28′ and 46°17′ north, Sibiu County displays a typical moderate temperate continental climate characterized by four distinct seasons. At latitudes above 35° north, the cutaneous vitamin D synthesis is less efficient than at lower latitudes [111,112]. Research has established a significant correlation between the vitamin D status and latitude in European countries with temperate continental climates [71]. In our study, both the monthly and seasonal distribution of the mean maternal 25(OH)D values were lower in winter and spring and higher in summer and autumn, and the infant’s values closely reflect the mother’s status (Figure 2 and Figure 3). Except for an unexpected increase in the maternal and neonatal 25(OH)D levels in November, the trend of the maternal and neonatal vitamin D status in our study was similar to the one described by other Romanian studies conducted in various regions of the country [70,71,72,74,75,113]. Similar seasonal variations in the population’s 25(OH)D values were found in the neighboring country, Hungary [114]. Our results are also consistent with data for Central Europe, with lower 25(OH)D values reported during the winter and higher values in the summer in children aged 0 to 9 years [115]. We observed the lowest maternal and neonatal mean 25(OH)D values in December and the highest in August. This contradicts the findings of Marti et al. [116] who reported minimum 25(OH)D values in March and maximum values in September. However, their study was performed in Arad County, a region with a warmer climate compared to Sibiu County. A study in the Bucharest area, another region with a warmer climate, also reported a maximum 25(OH)D value in September [72].

### 4.4. Maternal Vitamin D Status and Adverse Pregnancy Outcomes

Numerous studies have reported associations between maternal VDD and adverse pregnancy outcomes, yet the results vary across the research [6,9,12,16,17,22,56,57,58,59,60,61,62,63,64,65,66,67,68]. Several factors may prevent researchers from establishing a clear connection between the vitamin D status during pregnancy and pregnancy and birth outcomes, as suggested by a randomized controlled study conducted by Rostami et al. [117] involving 1763 pregnant women evaluated in the third trimester. The authors reported a variation in cut-off values for the maternal 25-hydroxyvitamin D (25(OH)D) status regarding the risk of gestational diabetes (highest risk at ≤6 ng/mL), preterm birth, and preeclampsia (highest risk at ≤15 ng/mL). The authors of this study recommended that vitamin D supplementation during pregnancy should aim for a 25(OH)D level > 15 ng/mL. Perhaps the most important challenge in establishing a causal relationship between suboptimal levels of vitamin D and various health conditions is that the different cut-off values used to define vitamin D sufficiency, VDI, VDD, and severe VDD are based on only low to moderate evidence of its extraskeletal effects [2,5,118]. Numerous studies have primarily focused on 25(OH)D levels recognized as adequate solely for bone health. However, there is a growing understanding that higher or alternative levels of 25(OH)D may be required to facilitate the various extraskeletal effects of vitamin D. Unfortunately, these optimal levels have yet to be clearly established. A recent theory criticizes the design of the studies on the vitamin D status, mentioning issues related to sample size, initiation, duration, daily dosed adjustments of vitamin D supplementation, various exposures to vitamin D through the diet or different types of vitamin D supplements, and potential biases in analyzing collected data, factors potentially influencing the studies’ results. Experts suggest that approaching vitamin D as a nutrient, with cut-off values for both beneficial and adverse effects, and assessments in studies with an observational design would be more appropriate than the current approach treating vitamin D as a drug [2,5,119]. In the preterm population, the gaps are even larger as there is no consensus on the cut-off values defining sufficient, deficient, or insufficient vitamin D levels. The current suggested values are primarily derived from the adult population and are likely inadequate for this population [42].

No significant risk for severe VDD, VDD, or VDI was found in pregnancies achieved through assisted reproductive techniques or in cases of multiple pregnancies (Table 3). Similarly to results reported by Christoph et al. [9], severe VDD, VDD, or VDI were not associated with an increased number of spontaneous abortions, assuming that the mothers had comparable vitamin D levels in previous pregnancies. Our results differ from those of Chen et al. [57], who noted a higher rate of spontaneous abortions associated with VDD; however, the VDD rate in their study was significantly greater than ours (83.28% compared to 55.48%). Although not statistically significant, the absence of surveillance during pregnancy by a family physician was linked to a higher incidence of maternal VDD, severe VDD, and VDI. Additionally, a lack of specialized obstetric care during pregnancy was correlated with an increased risk of VDD (RR 1.43) and VDI (RR 1.46) (Table 3). The social and educational background of the mothers included in our study may provide context for the inadequate prenatal care in a significant proportion of patients.

Despite being the most commonly encountered complications in mothers delivering at ≤32 weeks of gestation, we found no correlations between the maternal vitamin D status and imminent premature birth, hypertension, infections (all types), urinary tract infections, diabetes, or placental abnormalities (*p* > 0.05). Our findings contrast with other studies that have reported a significantly increased risk of high blood pressure during pregnancy associated with VDD [6,55,58,60,83]. The difference in results may be attributed to the considerably larger number of participants included in those studies than compared to ours. Numerous studies reported a significant association between gestational diabetes and VDD and even VDI [6,9,13,58,61,63,120], an association not found in our research. Notably, a Cochrane review by Palacios et al. [67] concluded that vitamin D supplementation during pregnancy probably decreases the gestational diabetes risk. We could not locate data on the association between VDD or VDI and imminent premature delivery (defined as uterine contractions associated with the dilatation and effacement of the uterine cervix) or placental abnormalities. However, a potential link between VDD or VDI and infections, including urinary tract infections, seems plausible given the well-documented anti-inflammatory effects of vitamin D [66,121,122]. The updated Cochrane review by Palacios et al. [67] indicates that vitamin D supplementation reduces the risk of severe postpartum hemorrhages. However, we did not find any references regarding a possible link between VDD and hemorrhages due to placental abnormalities in pregnancy. Interestingly, mothers with 25(OH)D levels greater than 20 ng/mL exhibited significantly higher rates of thyroid conditions. Currently, the relationship between the vitamin D status and thyroid function during pregnancy is not entirely understood [123,124], and our study had insufficient power to explore the association. In our study, we observed a relatively similar proportion of complications across all groups classified based on the 25(OH)D values as having a severe VDD, VDD, VDI, or sufficient vitamin D status, contradicting the findings reported by Alanazi et al. [82]. While our study reported comparable rates of VDD and VDI (59.8% versus 55.48%), their research included 228 mothers who delivered after 28 weeks of gestation without a vitamin D supplementation during pregnancy.

No significant association was found between the maternal vitamin D status, classified as severe VDD, VDD, or VDI, and the rates of fetal distress before delivery, prolonged preterm rupture of amniotic membranes, and delivery mode. Increased rates of emergency cesarean sections were reported in association with VDD by Alanazi et al. [82]. However, the Gallo et al. [66] meta-analysis found no link between higher levels of 25(OH)D following a maternal vitamin D supplementation in pregnancy and the delivery mode. Dullaert et al. [17] found no association between vitamin D statuses and surgical delivery. In a study on 3923 pregnant women, Cheng et al. [125] reported no causal relationship between the maternal vitamin D status and the preterm rupture of membranes or the prolonged preterm rupture of membranes. Two recent reviews concluded that a maternal vitamin D supplementation during pregnancy has unclear benefits. They highlighted the need for new studies after reviewing the targeted 25(OH)D levels and with the enrollment of the participants preconceptionally or at least in the first trimester of pregnancy or the initiation of vitamin D before conception given the important vitamin D effects in placenta formation and fetal development [126,127]. We also suspect that the low rate and inadequate dose of vitamin D supplementations in our study may have hindered our ability to find significant associations between the maternal vitamin D status and pregnancy outcomes.

### 4.5. Vitamin D Supplementation During Pregnancy and Its Effect on Maternal Vitamin D Status at Birth

The maternal data analysis revealed that a supplementation with multivitamins containing vitamin D may reduce the risk for severe VDD, VDD, and VDI (*p* = 0.001–0.006). Similar associations were observed with the use of specific vitamin D supplements (*p* < 0.001 to *p* = 0.035) (Table 2). The duration of the vitamin D supplementation did not significantly influence the vitamin D status. However, higher daily doses were significantly associated only with VDD (*p* = 0.014) and vitamin D sufficiency (*p* = 0.016) (Table 3). This may be attributed to a reduced rate of supplementation (43.15%), the various contents of vitamin D in the multivitamin used in pregnancy (from 200 IU to 800 IU), and the short period of the supplementation in all groups (median of 4 months). Notably, only one mother reported starting a vitamin D supplementation prenatally and continuing for 10 months. The majority of mothers did not use vitamin D for more than 6 months, and nearly all began supplementations after the second or third month of pregnancy. Only 16 mothers reported 25(OH)D screening during pregnancy as recommended by a physician.

### 4.6. Independent Risk Factor for Inadequate Maternal Vitamin D Status at Birth

Only increased parity and birth during the cold season (winter and spring) were independently associated with a severe VDD in the logistic regression analyses, and the predictive model built using these two variables demonstrated a good accuracy for severe VDD prediction (AUC 0.742; *p* < 0.001) (Figure 4). A maternal VDD (defined by 25(OH)D values < 20 ng/mL) at birth was independently associated with a lower maternal educational level, birth during the cold season, and absent administration of any vitamin D supplements. The predictive model developed using these risk factors yielded an AUC of 0.775; *p* < 0.001 (Figure 5). Furthermore, the absence of any vitamin D supplementation independently predicted VDI both in the logistic regression analysis and in a predictive model built using ROCs (AUC 0.794; *p* < 0.001). Our data suggest, similarly to other studies, that a lower social and educational status [82], birth during seasons with a reduced exposure to sunlight [8], and absence of a vitamin D supplementation [8,66,82] are important risk factors for a suboptimal maternal vitamin D status during pregnancy or at birth.

## 5. Final Comments

Our study has several limitations. First, it is an observational study, which carries inherent limitations associated with this design. As this is the first report from Romania regarding the vitamin D status in mothers who deliver very prematurely and their infants, we were unable to determine a sample size that would have provided this study with sufficient power to detect the impact of maternal vitamin D supplementations during pregnancy on pregnancy outcomes. We recognize that the exclusion criteria applied in our study reduced the number of participants and may have introduced a selection bias. However, we deemed it important to include only infants admitted within the first 24 h after birth to ensure consistent sampling timing. We applied similar criteria by excluding mothers who were admitted to our unit more than 48 h after delivery or who did not stay with us following the transfer of their preterm infant. Many studies on the neonatal vitamin D status excluded infants with major congenital anomalies or chromosomal abnormalities or those who died in the first 48 h of life. Applying the same exclusion criteria was important for comparing our results with the findings in other studies. Additionally, we felt it was ethically questionable to approach parents dealing with serious, life-threatening neonatal conditions for consent to participate in our study. Notably, all the mothers we approached expressed their willingness to participate.

We have not evaluated the effect of skin pigmentation, race, ethnicity, or the participants’ body weight, as this information is sensitive and not routinely collected. Due to the limited sample size and number of affected participants, we grouped conditions like hypertension, diabetes, and infections during pregnancy into a single category for analysis, although we collected data on specific conditions, including pre-existing hypertension, pregnancy-induced hypertension, preeclampsia, pre-existing diabetes, gestational diabetes, and chorioamnionitis. The small sample size may have interfered with the statistical analysis and results. Also, limited published information exists regarding the point-of-care method used for 25(OH)D analysis. We selected this method and standardized the timing of blood sampling to minimize the number of punctures and associated pain, reducing the risk of iatrogenic anemia in the preterm infants included in this study, as well as for financial reasons. Different definitions for a severe VDD, VDD, VDI, and sufficient vitamin D status complicate the comparison of results across various studies. Lastly, our data should be interpreted cautiously as they refers to a population in a specific region. A significant seasonal association with the maternal vitamin D status was demonstrated, and no other published data are available for comparison within a similar population. Our study’s primary strengths lie in its prospective cohort design and the standardized timing of the blood sampling, which facilitates a thorough evaluation of the vitamin D status in both mothers and their offspring.

## 6. Conclusions

Our study provides important epidemiological and clinical information for pregnant women delivering very prematurely, their preterm infants, professionals (such as family physicians, obstetricians, neonatologists, and pediatricians), and health policymakers. Additional regional and national data on vitamin D levels in pregnant women and preterm infants are essential to validate our study’s findings. Despite the National Guideline for the Evaluation and Therapy of Vitamin D Status in Pregnant Women, Newborns, and Children [76], since 2019, fewer than 50% of mothers delivering prematurely reported using vitamin D supplements during pregnancy. Most of these mothers initiated vitamin D supplementation late, typically towards the end of the first trimester of pregnancy or even later, and for only a short time, mostly using multivitamins that contained an inappropriate daily dosage of vitamin D. Vitamin D status screening is rarely recommended during pregnancy, and even in high-risk pregnancies, vitamin D supplements are not indicated based on 25(OH)D levels, as the national guideline recommends. The Romanian Ministry of Health, along with all professional societies involved in the care of pregnant women and neonates, should consider interventions to improve the vitamin D status of mothers and newborns and strategies to promote and reinforce the current national guidelines.

In line with the existing literature, we found a significant correlation between maternal and neonatal 25(OH)D levels and between maternal and neonatal severe VDD, VDD, and VDI in the case of preterm deliveries at ≤32 weeks of gestation. Preterm infants with a GA of 32 weeks or less are particularly vulnerable. They may face increased risks for short- and long-term complications related to prematurity, including respiratory issues, infections, neurological problems, neurodevelopmental delays, and impaired growth and development. It is crucial to acknowledge that this population of neonates is at a heightened risk for developmental and neurological complications due to their increased immaturity at birth. Improving their vitamin D status at birth may reduce the risk of multiple complications associated with prematurity, according to the recent data in the literature. It would be valuable to conduct a replication of this study focusing on pregnant women who deliver late preterm and at-term infants, along with their offspring. Such research would provide essential insights for obstetrical and neonatal practices, as well as inform healthcare policies. It is important to determine whether vitamin D deficiency is confined to mothers of very preterm infants or if it affects the broader population of pregnant women. To enhance vitamin D levels in both mothers and their fetuses, it is essential for all healthcare professionals involved in pregnancy care to consistently adhere to national guidelines regarding vitamin D supplementation. Additionally, screening for vitamin D status at birth and providing personalized vitamin D supplementations as soon as possible for preterm infants could help enhance both short-term and long-term health outcomes for this vulnerable group.

## Figures and Tables

**Table 1:** Maternal and neonatal characteristics of the study group.

Maternal Characteristics (N = 146)	
Age (years) (mean ± SD) (range)	29.33 ± 6.89 (14–47)
Urban residence (N/%)	52 (35.62)
Marital status—married (N/%)	87 (59.59)
Educational level	
Illiterate (N/%)	3 (2.05)
Primary school (N/%)	45 (30.82)
College (N/%)	50 (34.25)
High school (N/%)	48 (32.88)
Number of gestations (mean ± SD) (range)	2.82 ± 2.16 (1–12)
Parity (mean ± SD) (range)	2.10 ± 1.37 (1–8)
Pregnancies obtained using assisted reproductive techniques (N/%)	7 (4.79)
Multiple pregnancy (N/%)	21 (14.38)
Follow-up at the family physician (N/%)	125 (85.62)
Follow-up at the obstetrician (N/%)	100 (68.49)
Pregnancy complications—all (N/%) ^&^	100 (68.9)
Imminent preterm delivery (n/%)	37 (25.34)
Hypertension (N/%)	25 (17.12)
Infections—all types (N/%)	25 (17.12)
Urinary tract infections (N/%)	15 (10.27)
Thyroid conditions (N/%)	12 (8.22)
Placental abnormalities (N/%)	9 (6.16)
Diabetes (N/%)	5 (3.42)
Asthma (N/%)	2 (1.37)
Fetal distress (N/%)	8 (5.48)
PPROM over 18 h (N/%)	31 (21.23)
C-section delivery (N/%)	62 (42.46)
Season of birth	
Winter (December, January, February) (N/%)	42 (28.77)
Spring (March, April, May) (N/%)	31 (21.23)
Summer (June, July, August) (N/%)	39 (26.71)
Autumn (September, October, November) (N/%)	34 (23.29)
Cold season (winter + spring) (N/%)	73 (50.00)
Special diet during pregnancy (N/%)	1 (0.68)
Vitamin D supplementation during pregnancy	
Any type of vitamin D supplements (N/%)	63 (43.15)
Vitamin D supplements (N/%)	16 (10.96)
Vitamin D supplementation duration (months) (mean ± SD) (range)	4.12 ± 1.66 (1–10)
Vitamin D supplementation dose/day (IU) (mean ± SD) (range)	907.55 ± 806.1 (100–4000)
Maternal 25(OH)D status (ng/mL) (mean ± SD) (range)	20.96 ± 12.57 (4.2–69.0)
**Neonatal Characteristics (N = 164)**	
Gestational age (weeks) (mean ± SD) (range)	28.93 ± 5.63 (23–32)
Birth weight (g) (mean ± SD) (range)	1225.95 ± 399.21 (475–2110)
Apgar score at 1 min (mean ± SD) (range)	6.89 ± 1.74 (1–9)
Apgar score at 5 min (mean ± SD) (range)	7.51 ± 1.29 (3–10)
Neonatal 25(OH)D status (ng/mL) (mean ± SD) (range)	20.13 ± 11.05 (5–70.4)

Legend: N—number, SD—standard deviation, IU—international units; and ^&^—rare complications not included in the analysis: renal diseases and asthma.

**Table 2:** Correlations between maternal vitamin D supplementation during pregnancy and neonatal vitamin D status with maternal and neonatal vitamin D status, gestational age, and birth weight *.

	Maternal Vitamin D Status			
	R	r^2^	*p*	B
Duration of vitamin D supplementation (months)	0.304	0.092	0.016	2.48
Vitamin D dose/day (IU/day)	0.344	0.119	0.012	20.31
Neonatal 25(OH)D	0.684	0.468	<0.001	0.62
Gestational age (weeks)	−0.185	0.035	0.025	−0.039
Birth weight (g)	−0.133	0.018	0.109	−4.122
	Neonatal vitamin D status			
Duration of maternal vitamin D supplementation (months)	0.385	0.148	0.002	2.95
Vitamin D dose/day during pregnancy (IU/day)	0.535	0.286	<0.001	0.01
Gestational age (weeks)	0.065	0.004	0.408	0.273
Birth weight (g)	0.031	0.001	0.690	0.001

Legend: * linear regression.

**Table 3:** Associations between maternal characteristics and pregnancy outcomes with severe vitamin D deficit (25(OH)D <10 ng/mL), deficit (25(OH)D < 20 ng/mL), and insufficiency (25(OH)D 20–29 ng/mL).

	Maternal 25(OH)D Values								
	<10 ng/mL vs. ≥10 ng/mL			<20 ng/mL vs. ≥20 ng/mL			20–29 ng/mL vs. ≥30 ng/mL		
	<10 N = 29	>10 N = 117	*p*/RR (95% CI)	<20 N = 81	>20 N = 65	*p*/RR (95% CI)	20–29 N = 116	>30 N = 30	*p*/RR (95% CI)
Maternal age (years) (mean ± SD)	29.21 ± 6.84	29.36 ± 6.93	0.916 *	29.01 ± 7.29	29.72 ± 6.38	0.537 *	24.27 ± 2.94	40.57 ± 9.98	<0.001 *
Gestations (mean ± SD)	3.72 ± 2.94	2.60 ± 1.87	0.012 *	3.28 ± 2.62	2.26 ± 1.24	0.005 *	2.29 ± 1.30	2.23 ± 1.19	0.867 *
Parity (mean ± SD)	2.69 ± 1.90	1.95 ± 1.17	0.009 *	2.41 ± 1.61	1.69 ± 0.86	0.001 *	1.83 ± 0.92	1.53 ± 0.78	0.172 *
Abortions (mean ± SD)	1.07 ± 2.02	0.63 ± 1.24	0.142 *	0.85 ± 1.76	0.57 ± 0.88	0.243 *	0.46 ± 0.82	0.70 ± 0.95	0.273 *
Education status (N/%)									
Illiterate	2/6.9	1/0.9	0.040 **	2/2.5	1/1.5	<0.001 **	1/2.1	0/0	0.266 **
Primary school	11/37.9	34/29.1		33/40.0	12/18.5		9/25.7	3/10.0	
College	10/34.5	40/34.2		30/37.0	20/30.8		8/22.9	12/40.0	
Highschool	6/20.7	42/35.9		16/19.8	32/49.2		17/48.6	15/50.0	
Urban residence (N/%)	7/24.1	45/38.5	0.151 ** RR 0.63 (0.32–1.24)	24/29.6	28/43.1	0.093 ** RR 0.69 (0.44–1.06)	13/37.1	15/50.0	0.304 ** RR 0.78 (0.48–1.26)
Marital status: unmarried (N/%)	16/55.2	43/36.8	0.071 ** RR 1.50 (1.00–2.25)	40/49.4	19/29.2	0.013 ** RR 1.69 (1.09–2.62)	12/34.3	7/23.3	0.341 ** RR 1.26 (0.81–1.98)
Season of birth (N/%)									
Winter	17/58.7	25/21.4	<0.001 **	31/38.3	11/16.9	0.001 **	5/14.3	6/20.0	0.280 **
Spring	5/17.2	26/22.2		21/25.9	10/15.4		5/14.3	5/16.7	
Summer	3/10.3	36/30.8		13/16.0	26/40.0		13/37.1	13/43.3	
Autumn	4/13.8	30/25.6		16/19.8	18/27.7		212/34.3	6/20.0	
Cold season (vs. warm season)	22/75.9	51/43.6	0.002 ** RR 1.74 (1.30–2.33)	52/64.2	21/32.3	<0.001 ** RR 1.98 (1.35–2.93)	10/28.6	11/36.7	0.494 ** RR 0.84 (0.50–1.41)
ART pregnancies (N/%)	1/3.4	6/5.1	0.707 ** RR 0.67 (0.08–5.37)	1/1.2	6/9.2	0.025 ** RR 0.13 (0.02–1.08)	2/5.7	4/13.3	0.297 ** RR 0.66 (0.35–1.25)
Multiple pregnancies (N/%)	4/13.8	17/14.5	0.920 ** RR 0.95 (0.35–2.61)	9/11.1	12/18.5	0.211 ** RR 0.60 (0.27–1.34)	6/17.1	6/20.0	0.772 ** OR 0.91 (0.48–1.71)
No primary care follow-up (N/%)	5/17.2	16/13.7	0.627 ** OR 1.26 (0.50–3.16)	14/17.5	7/10.8	0.268 ** OR 1.25 (0.89–1.77)	5/14.3	2/6.7	0.179 ** RR 2.46 (0.60–9.97)
No obstetrical controls (N/%)	12/41.4	34/29.1	0.204 ** OR 0.89 (0.73–1.08)	32/40.0	14/21.5	0.020 ** OR 1.43 (1.09–1.90)	10/28.6	4/13.3	0.141 ** OR 1.46 (0.94–2.25)
Pregnancy complications (N/%)									
Diabetes	3/10.3	2/1.7	0.022 ** RR 6.05 (1.04–34.5)	3/3.7	2/3.1	0.827 ** RR 1.20 (0.21–6.99)	0/0	2/6.7	0.125 **
Hypertension	7/24.1	18/15.4	0.266 ** RR 1.60 (0.72–3.40)	14/17.3	11/16.9	0.955 ** RR 1.02 (0.50–2.10)	7/20.0	4/13.3	0.483 ** RR 1.32 (0.58–3.03)
Thyroid disorders	2/6.9	10/8.5	0.774 ** RR 0.80 (0.91–1.14)	3/3.7	9/13.8	0.027 ** RR 0.27 (0.07–0.95)	2/5.7	7/23.3	0.041 ** RR 0.53 (0.33–0.84)
Urinary tract infections	1/3.4	14/12	0.179 ** RR 0.29 (0.04–2.10)	6/7.4	9/13.8	0.205 ** RR 0.53 (0.20–1.43)	5/14.3	4/13.3	0.913 ** RR 1.04 (0.48–2.28)
All infections	4/13.8	21/17.9	0.598 ** RR 0.77 (0.29–2.07)	12/14.8	13/20.0	0.412 ** RR 0.74 (0.36–1.51)	6/17.1	7/23.3	0.541 ** RR 0.82 (0.46–1.48)
Imminent preterm birth	7/24.1	30/25.6	0.869 ** RR 0.94 (0.46–1.92)	18/22.2	19/29.2	0.337 ** RR 0.76 (0.44–1.33)	8/22.9	11/36.7	0.229 ** RR 0.71 (0.43–1.19)
Placental anomalies	0/0	9/7.7	0.125 **	4/4.9	5/7.7	0.495 ** RR 0.64 (0.18–2.29)	2/5.7	3/10.0	0.525 ** RR 0.75 (0.35–1.62)
All pregnancy complications	18/62.1	82/70.1	0.409 ** RR 0.89 (0.65–1.20)	51/64.2	48/73.8	0.205 ** RR 0.87 (0.70–1.08)	25/71.4	23/76.7	0.638 ** RR 1.13 (0.70–1.83)
Fetal distress (N/%)	0/0	8/6.8	0.150 **	5/6.2	3/4.6	0.684 ** RR 1.34 (0.33–5.39)	1/2.9	2/6.7	0.473 ** RR 0.68 (0.29–1.58)
PPROM > 18 h (N/%)	3/10.3	28/23.9	0.111 ** RR 0.43 (0.14–1.32)	15/18.5	16/24.6	0.374 ** RR 0.75 (0.40–1.40)	10/28.6	6/20.0	0.432 ** RR 1.31 (0.62–2.61)
Cesarean section (N/%)	13/44.8	49/41.9	0.776 ** RR 1.07 (0.68–1.69)	36/44.4	26/40.0	0.592 ** RR 1.11 (0.76–1.63)	16/45.7	10/38.5	0.317 ** RR 1.33 (0.75–2.37)
Vitamin D supplementation									
Multivitamins containing vitamin D (N/%)	6/20.7	57/48.7	0.006 ** RR 0.42 (0.20–0.89)	24/30.9	38/58.5	0.001 ** RR 0.53 (0.36–0.77)	14/40.0	24/80.0	0.001 ** RR 0.35 (0.17–0.74)
Vitamin D supplements (N/%)	0/0	16/13.7	0.035 **	1/1.2	15/23.1	<0.001 ** RR 0.05 (0.01–0.39)	2/5.7	13/43.3	<0.001 ** RR 0.39 (0.25–0.61)
Duration (months) (median ± IQR)	4.5 (4–5.25)	4(3–5)	0.191 ^	4 (3–4.5)	4(3–6)	0.126 ^	4(3–5)	4.5(3–6)	0.191 ^
Daily vitamin D dose (IU/day) (median ± IQR)	600 (287–800)	800 (250–2000)	0.733 ^	250 (200–800)	800 (300–2000)	0.014 ^	600 (200–800)	1000 (800–2000)	0.016 ^

Legend: N—number, SD—standard deviation, RR—risk ratio, CI—confidence interval, *—Independent Samples *T* Test, **—chi square test, ^—Mann–Whitney U Test, IU—international units, PPROM—preterm premature rupture of membranes, and IQR—interquartile range.

**Table 4:** Associations between maternal severe vitamin D deficit (25(OH)D < 10 ng/mL), deficit (25(OH)D < 20 ng/mL), and insufficiency (25(OH)D 20–29 ng/mL) and neonatal characteristics.

	Maternal 25(OH)D Status								
	<10 ng/mL vs. ≥10 ng/mL			<20 ng/mL vs. ≥20 ng/mL			20–29 ng/mL vs. ≥30 ng/mL		
	<10 N = 29	>10 N = 117	*p*/RR (95% CI)	<20 N = 81	>20 N = 65	*p*/RR (95% CI)	20–29 N = 116	>30 N = 30	*p*/RR (95% CI)
Neonatal 25OHD values (ng/mL) (mean ± SD)	10.10 ± 4.71	22.65 ± 10.75	<0.001 *	13.80 ± 6.00	27.64 ± 10.99	<0.001 *	10.57 ± 9.51	32.85 ± 11.38	0.002 *
BW (mean ± SD)	1220 ± 325.18	1226.95 ± 412.07	0.928 *	1238.15 ± 402.74	1210.60 ± 388.26	0.658 *	1192.29 ± 366.52	1163.17 ± 421.08	0.767 *
GA (median ± IQR)	30 (28–31)	30 (27–31)	0.974 ^	30 (27.5–31)	30 (27–31)	0.461 ^	30 (27–31)	30 (25–31)	0.168 ^
Male gender (N/%)	21/63.6	73/55.7	0.415 ** RR 1.14 (0.85–1.54)	50/56.2	44/58.7	0.750 ** RR 0.96 (0.73–1.25)	24/57.1	20/60.6	0.766 ** RR 0.94 (0.63–1.40)
Apgar at 1 min (median ± IQR)	7 (6–8)	7 (6–8)	0.861 ^	7 (6–8)	7 (6–8)	0.583 ^	7 (6–8)	7 (6–8)	0.616 ^
5 min Apgar (median ± IQR)	8 (7–9)	8 (7–8)	0.518 ^	8 (7–7.5)	8 (6–8)	0.257 ^	8 (6–8)	8 (7–8)	0.821 ^
Severe neonatal 25(OH)D deficit (<10 ng/mL) (N/%)	19/57.6	8/6.1	<0.001 ** RR 9.43 (4.53–19.61)	25/28.1	2/2.7	<0.001 ** RR 10.53 (2.58–43.02)	2/4.8	0	0.209 **
Vitamin D status									
≤10 ng/mL	19/57.6	8/6.1	<0.001 **	25/8.1	2/2.7	<0.001 **	2/4.8	0	0.039 **
10–19 ng/mL	13/39.4	56/42.7		52/58.4	17/22.7		12/28.6	5/15.2	
20–29 ng/mL	1/3	41/31.3		11/12.4	31/41.3		17/40.5	14/42.4	
≥30 ng/mL	0/0	26/19.8		1/1.1	25/33.3		11/26.2	14/42.4	

Legend: N—number, RR—risk ratio, CI—confidence interval, SD—standard deviation, BW—birth weight, GA—gestational age, *—Independent Samples *T* Test, **—chi square test, ^—Mann–Whitney U Test, and IQR—interquartile range.

**Table 5:** Multivariable regression to identify independent risk factors for severe vitamin D deficiency (25(OH)D < 10 ng/mL).

Variable *	B	Wald	*p*	OR (95% CI)
Cold season	1.328	7.523	0.003	3.775 (1.461–9.752)
Lower number of births (parity)	−0.381	5.837	0.016	0.683 (0.501–0.931)
PPROM > 18 h	1.270	3.261	0.071	3.562 (0.897–14.137)

Legend: PPROM—preterm premature rupture of membranes; * variables with a *p* value < 0.200 in the univariate analysis were introduced in the binary logistic regression: maternal residence, marital status, gestational age, number of gestations, complications during pregnancy, placental abnormalities, supplementation with any type of vitamin D supplements, and vitamin D supplementation were excluded step by step.

**Table 6:** Multivariable regression to identify independent risk factors for vitamin D deficiency (25(OH)D < 20 ng/mL).

Variable *	B	Wald	*p*	OR (95% CI)
Higher educational level	−1.120	7.140	0.008	0.326 (0.143–0.742)
Cold season	1.324	14.122	<0.001	3.757 (1.884–7.493)
Supplementation with vitamin D supplements	−3.030	7.801	0.005	0.048 (0.006–0.405)

Legend: * variables with a *p* value < 0.200 in the univariate analysis were introduced in the binary logistic regression (educational level, maternal residence, marital status, gestational age, number of gestations, parity, natural versus ART pregnancy, complications during pregnancy, thyroid diseases, pregnancy follow-up by obstetrician, and supplementation with any type of vitamin D supplements were excluded step by step); ART—assisted reproductive techniques.

**Table 7:** Multivariable regression to identify independent risk factors for vitamin D insufficiency (25(OH)D20–29 ng/mL).

Variable *	B	Wald	*p*	OR (95% CI)
Any supplements containing vitamin D during pregnancy	−1.166	3.498	0.061	0.312 (0.092–1.058)
Supplementation using vitamin D supplements	−1.959	5.108	0.024	0.141 (0.026–0.771)

Legend: * variables with a *p* value < 0.200 in the univariate analysis were introduced in the binary logistic regression: maternal age, parity, thyroid diseases, diabetes during pregnancy, pregnancy follow-up by family physician, and pregnancy follow-up by an obstetrician were excluded step by step.

## Data Availability

The data presented in this study are available on request from the corresponding author. The data are not publicly available due to restrictions privacy.

## References

[1] van der Pligt P., Willcox J., Szymlek-Gay E.A., Murray E., Worsley A., Daly R.M. (2018). Associations of Maternal Vitamin D Deficiency with Pregnancy and Neonatal Complications in Developing Countries: A Systematic Review. Nutrients.

[2] Grant W.B., Wimalawansa S.J., Pludowski P., Cheng R.Z. (2025). Vitamin D: Evidence-Based Health Benefits and Recommendations for Population Guidelines. Nutrients.

[3] Roth D.E., Abrams S.A., Aloia J., Bergeron G., Bourassa M.W., Brown K.H., Calvo M.S., Cashman K.D., Combs G., De-Regil L.M. (2018). Global prevalence and disease burden of vitamin D deficiency: A roadmap for action in low- and middle-income countries. Ann. N. Y. Acad. Sci..

[4] Cui X., Fu J. (2023). Early prediction of bronchopulmonary dysplasia: Can noninvasive monitoring methods be essential?. ERJ Open Res..

[5] Wimalawansa S.J. (2023). Physiological Basis for Using Vitamin D to Improve Health. Biomedicines.

[6] Mansur J.L., Oliveri B., Giacoia E., Fusaro D., Costanzo P.R. (2022). Vitamin D: Before, during and after Pregnancy: Effect on Neonates and Children. Nutrients.

[7] Liu Y., Ding C., Xu R., Wang K., Zhang D., Pang W., Tu W., Chen Y. (2022). Effects of vitamin D supplementation during pregnancy on offspring health at birth: A meta-analysis of randomized controlled trails. Clin. Nutr..

[8] Bärebring L., Amberntsson A., Augustin H. (2022). A validated screening tool correctly identifies the majority of pregnant women at high risk of vitamin D deficiency. Clin. Nutr. ESPEN.

[9] Christoph P., Challande P., Raio L., Surbek D. (2020). High prevalence of severe vitamin D deficiency during the first trimester in pregnant women in Switzerland and its potential contributions to adverse outcomes in the pregnancy. Swiss Med. Wkly..

[10] Monangi N., Slaughter J.L., Dawodu A., Smith C., Akinbi H.T. (2014). Vitamin D status of early preterm infants and the effects of vitamin D intake during hospital stay. Arch. Dis. Child. Fetal Neonatal Ed..

[11] Mendes M.M., Darling A.L., Hart K.H., Morse S., Murphy R.J., Lanham-New S.A. (2019). Impact of high latitude, urban living and ethnicity on 25-hydroxyvitamin D status: A need for multidisciplinary action?. J. Steroid Biochem. Mol. Biol..

[12] World Health Organization (2020). WHO Antenatal Care Recommendations for a Positive Pregnancy Experience: Nutritional Interventions Update: Vitamin D Supplements During Pregnancy.

[13] Agüero-Domenech N., Jover S., Sarrión A., Baranda J., Quesada-Rico J.A., Pereira-Expósito A., Gil-Guillén V., Cortés-Castell E., García-Teruel M.J. (2021). Vitamin D Deficiency and Gestational Diabetes Mellitus in Relation to Body Mass Index. Nutrients.

[14] Zung A., Topf-Olivestone C., Shinwell E.S., Hofi L., Juster-Reicher A., Flidel-Rimon O. (2020). Reassessing vitamin D supplementation in preterm infants: A prospective study and review of the literature. J. Pediatr. Endocrinol. Metab..

[15] Matejek T., Zemankova J., Malakova J., Cermakova E., Skalova S., Palicka V. (2022). Severe vitamin D deficiency in preterm infants: Possibly no association with clinical outcomes?. Matern. Fetal Neonatal Med..

[16] You Z., Mei H., Zhang Y., Song D., Zhang Y., Liu C. (2024). The effect of vitamin D deficiency during pregnancy on adverse birth outcomes in neonates: A systematic review and meta-analysis. Front. Pediatr..

[17] Dullaert B., Schroven S., Jacquemyn Y. (2018). The effect of maternal vitamin D status on pregnancy outcome and child health in the first year of life. Clin. Exp. Obstet. Gynecol..

[18] Matejek T., Navratilova M., Zaloudkova L., Malakova J., Maly J., Skalova S., Palicka V. (2020). Vitamin D status of very low birth weight infants at birth and the effects of generally recommended supplementation on their vitamin D levels at discharge. J. Matern. Fetal Neonatal Med..

[19] Baldan E., Yarcı E. (2022). Serum 25-Hydroxyvitamin D Levels in Preterm Infants Born at Gestational Age of ≤32 Weeks and Prematurity-related Morbidities and Complications. J. Dr. Behcet Uz Child. Hosp..

[20] GhehsarehArdastani A., Hashemi E., Beheshtinejad M., Dorostkar R. (2020). Comparison of 25- Hydroxy Vitamin D Levels in Premature Infants with and without Respiratory Distress. Iran. J. Neonatol..

[21] Boskabadi H., Mamoori G., Khatami S.F., Faramarzi R. (2018). Serum level of vitamin D in preterm infants and its association with premature-related respiratory complications: A case-control study. Electron. Physician..

[22] Yu H., Fu J., Feng Y. (2022). Utility of umbilical cord blood 25-hydroxyvitamin D levels for predicting bronchopulmonary dysplasia in preterm infants with very low and extremely low birth weight. Front. Pediatr..

[23] Zhang X., Luo K., He X., Chen P. (2021). Association of vitamin D status at birth with pulmonary disease morbidity in very preterm infants. Pediatr. Pulmonol..

[24] Papalia H., Samonini A., Buffat C., Gras E., des Robert C., Landrier J.F., Pauly V., Boubred F. (2022). Low Vitamin D Levels at Birth and Early Respiratory Outcome in Infants With Gestational Age Less Than 29 Weeks. Front. Pediatr..

[25] Budič P., Paro-Panjan D., Duh K., Soltirovska-Šalamon A. (2022). The influence of maternal levels of vitamin D and adiponectin on offspring’s health. Pediatr. Neonatol..

[26] Nabiel N., Nugroho H.W., Moelyo A.G. (2024). Vitamin D Deficiency is Associated with Hypocalcemia in Preterm Infants. Mol. Cell. Biomed. Sci..

[27] Park H.W., Lim G., Park Y.-M., Chang M., Son J.S., Lee R. (2020). Association between vitamin D level and bronchopulmonary dysplasia: A systematic review and meta-analysis. PLoS ONE.

[28] Holick M.F., Binkley N.C., Bischoff-Ferrari H.A., Gordon C.M., Hanley D.A., Heaney R.P., Murad M.H., Weaver C.M., Endocrine Society (2011). Evaluation, treatment, and prevention of vitamin D deficiency: An Endocrine Society clinical practice guideline. J. Clin. Endocrinol. Metab..

[29] Demay M.B., Pittas A.G., Bikle D.D., Diab D.L., Kiely M.E., Lazaretti-Castro M., Lips P., Mitchell D.M., Murad M.H., Powers S. (2024). Vitamin D for the Prevention of Disease: An Endocrine Society Clinical Practice Guideline. J. Clin. Endocrinol. Metab..

[30] Gioxari A., Papandreou P., Daskalou E., Kaliora A.C., Skouroliakou M. (2024). Association of Serum Calcium Levels of Preterm Neonates at Birth with Calcium Intake from Foods and Supplements by Bedridden Women during Pregnancy. Healthcare.

[31] Phiri C.B., Davis C.R., Grahn M., Gannon B.M., Kokinos B.P., Crenshaw T.D., Tanumihardjo S.A. (2024). Vitamin D Maintains Growth and Bone Mineral Density against a Background of Severe Vitamin A Deficiency and Moderate Toxicity in a Swine Model. Nutrients.

[32] Giustina A., Bilezikian J.P., Adler R.A., Banfi G., Bikle D.D., Binkley N.C., Bollerslev J., Bouillon R., Brandi M.L., Casanueva F.F. (2024). Consensus Statement on Vitamin D Status Assessment and Supplementation: Whys, Whens, and Hows. Endocr. Rev..

[33] Romero-Lopez M., Naik M., Holzapfel L.F., Tyson J.E., Pedroza C., Ahmad K.A., Rysavy M.A., Carlo W.A., Zhang Y., Tibe C. (2024). Enteral nutritional practices in extremely preterm infants: A survey of U.S. NICUs. J. Perinatol..

[34] Çetinkaya M., Çekmez F., Erener-Ercan T., Buyukkale G., Demirhan A., Aydemir G., Aydin F.N. (2015). Maternal/neonatal vitamin D deficiency: A risk factor for bronchopulmonary dysplasia in preterms?. J Perinatol..

[35] Boskabadi H., Zakerihamidi M., Mehrad-Majd H., Ghoflchi S. (2024). Evaluation of vitamin D in the diagnosis of infants with respiratory distress, the clinical value: A systematic review and *meta*-analysis. Paediatr. Respir. Rev..

[36] Salas A.A., Woodfin T., Phillips V., Peralta-Carcelen M., Carlo W.A., Ambalavanan N. (2018). Dose-Response Effects of Early Vitamin D Supplementation on Neurodevelopmental and Respiratory Outcomes of Extremely Preterm Infants at 2 Years of Age: A Randomized Trial. Neonatology.

[37] Yates N., Gunn A.J., Bennet L., Dhillon S.K., Davidson J.O. (2021). Preventing Brain Injury in the Preterm Infant-Current Controversies and Potential Therapies. Int. J. Mol. Sci..

[38] Ma S.S., Zhu D.M., Yin W.J., Hao J.H., Huang K., Tao F.B., Tao R.X., Zhu P. (2021). The role of neonatal vitamin D in the association of prenatal depression with toddlers ADHD symptoms: A birth cohort study. J. Affect. Disord..

[39] Sava F., Treszl A., Hajdú J., Toldi G., Rigó J., Tulassay T., Vásárhelyi B. (2016). Plasma vitamin D levels at birth and immune status of preterm infants. Immunobiology.

[40] Cutuli S.L., Ferrando E.S., Cammarota F., Franchini E., Caroli A., Lombardi G., Tanzarella E.S., Grieco D.L., Antonelli M., De Pascale G. (2024). Update on vitamin D role in severe infections and sepsis. J. AnesthAnalg Crit. Care.

[41] Fort P., Salas A.A., Nicola T., Craig C.M., Carlo W.A., Ambalavanan N. (2016). A Comparison of 3 Vitamin D Dosing Regimens in Extremely Preterm Infants: A Randomized Controlled Trial. J. Pediatr..

[42] Radu I.A., Ognean M.L., Ștef L., Giurgiu D.I., Cucerea M., Gheonea C. (2025). Vitamin D: What We Know and What We Still Do Not Know About Vitamin D in Preterm Infants—A Literature Review. Children.

[43] Reyes M.L., Vizcaya C., Le Roy C., Loureiro C., Brinkmann K., Arancibia M., Campos L., Iturriaga C., Pérez-Mateluna G., Rojas-McKenzie M. (2024). Weekly Vitamin D Supplementation to Prevent Acute Respiratory Infections in Young Children at Different Latitudes: A Randomized Controlled Trial. J. Pediatr..

[44] Yang Y., Li Z., Yan G., Jie Q., Rui C. (2018). Effect of different doses of vitamin D supplementation on preterm infants—An updated meta-analysis. J. Matern. Fetal Neonatal Med..

[45] Sanlier N., Guney-Coskun M. (2022). Vitamin D, the immune system, and its relationship with diseases. Egypt. Pediatr. Assoc. Gaz..

[46] Sassi F., Tamone C., D’Amelio P. (2018). Vitamin D: Nutrient, Hormone, and Immunomodulator. Nutrients.

[47] Sailike B., Onzhanova Z., Akbay B., Tokay T., Molnár F. (2024). Vitamin D in Central Nervous System: Implications for Neurological Disorders. Int. J. Mol. Sci..

[48] Sotunde O.F., Laliberte A., Weiler H.A. (2019). Maternal risk factors and newborn infant vitamin D status: A scoping literature review. Nutr. Res..

[49] Wagner C.L., Hollis B.W. (2022). The extraordinary metabolism of vitamin D. eLife.

[50] Ashley B., Simner C., Manousopoulou A., Jenkinson C., Hey F., Frost J.M., Rezwan F.I., White C.H., Lofthouse E.M., Hyde E. (2022). Placental uptake and metabolism of 25(OH)vitamin D determine its activity within the fetoplacental unit. eLife.

[51] Jutell M., Bhat S., Bagge M.L., Isberg P.E., Wiberg N. (2024). Correlation between maternal and umbilical cord 25-hydroxy-vitamin D levels over a range of values. A prospective observational study from the United Arab Emirates. PLoS ONE.

[52] Tofe-Valera I., Pérez-Navero J.L., Caballero-Villarraso J., Cañete M.D., Villa-Jiménez R., De la Torre-Aguilar M.J. (2023). Vitamin d deficiency with high parathyroid hormone levels is related to late onset SEPSIS among preterm infants. BMC Pregnancy Childbirth.

[53] Mori J.D., Kassai M.S., Lebrão C.W., Affonso-Fonseca F.L., Sarni R.O.S., Suano-Souza F.I. (2023). Influence of umbilical cord vitamin D serum levels on the growth of preterm infants. Nutrition.

[54] Keskinsoy B., Mutlu Sütçüoğlu B., Özdemir H., Bayram M. (2023). Vitamin D levels in pregnancies and neonatal outcomes. Perinat. J..

[55] Hollis B.W., Wagner C.L. (2022). Substantial Vitamin D Supplementation Is Required during the Prenatal Period to Improve Birth Outcomes. Nutrients.

[56] Zhang H., Wang S., Tuo L., Zhai Q., Cui J., Chen D., Xu D. (2022). Relationship between Maternal Vitamin D Levels and Adverse Outcomes. Nutrients.

[57] Chen G.D., Pang T.T., Li P.S., Zhou Z.X., Lin D.X., Fan D.Z., Guo X.L., Liu Z.P. (2020). Early pregnancy vitamin D and the risk of adverse maternal and infant outcomes: A retrospective cohort study. BMC Pregnancy Childbirth.

[58] Tammo Ö., Yıldız S. (2022). Vitamin D Deficiency and Its Clinical Results in Preeclamptic Mothers and Their Babies. Cureus.

[59] Yue C.Y., Gao J.P., Zhang C.Y., Ying C.M. (2021). Is serum vitamin D deficiency before gestational 20 weeks a risk factor for preeclampsia?. Clin. Nutr..

[60] Tous M., Villalobos M., Iglesias-Vázquez L., Fernández-Barrés S., Arija V. (2020). Vitamin D status during pregnancy and offspring outcomes: A systematic review and meta-analysis of observational studies. Eur. J. Clin. Nutr..

[61] Santos H.G.D., Longoni A., Trettim J.P., Lemes I.T., Menchaca J.C., do Amaral C.C., de Matos M.B., Quevedo L.A., Nedel F., Ghisleni G. (2024). Deficiency of vitamin D is associated with antenatal depression: A cross-sectional study. Trends Psychiatry Psychother..

[62] Zhao R., Zhou L., Wang S., Yin H., Yang X., Hao L. (2022). Effect of maternal vitamin D status on risk of adverse birth outcomes: A systematic review and dose-response meta-analysis of observational studies. Eur. J. Nutr..

[63] Kurmangali Z., Abdykalykova B., Kurmangali A., Zhantagulov D., Terzic M. (2024). The influence of vitamin D on pregnancy and outcomes: Current knowledge and future perspectives. Gynecol. Obstet. Investig..

[64] Benaim C., Carrilho T.R.B., Farias D.R., Kac G. (2021). Vitamin D during pregnancy and its association with birth outcomes: A Brazilian cohort study. Eur. J. Clin. Nutr..

[65] Treiber M., Mujezinović F., PečovnikBalon B., Gorenjak M., Maver U., Dovnik A. (2020). Association between umbilical cord vitamin D levels and adverse neonatal outcomes. J. Int. Med. Res..

[66] Gallo S., McDermid J.M., Al-Nimr R.I., Hakeem R., Moreschi J.M., Pari-Keener M., Stahnke B., Papoutsakis C., Handu D., Cheng F.W. (2020). Vitamin D Supplementation during Pregnancy: An Evidence Analysis Center Systematic Review and Meta-Analysis. J. Acad. Nutr. Diet..

[67] Palacios C., Kostiuk L.L., Cuthbert A., Weeks J. (2024). Vitamin D supplementation for women during pregnancy. Cochrane Database Syst. Rev..

[68] Wang S., Xin X., Luo W., Mo M., Si S., Shao B., Shen Y., Cheng H., Yu Y. (2021). Association of vitamin D and gene variants in the vitamin D metabolic pathway with preterm birth. Nutrition.

[69] Fang X., Xie Y., Cao S., Liu J., Shi Y., Yu L., Zheng T., Liu H., Li Y., Xu S. (2024). Associations between maternal urinary rare earth elements during pregnancy and birth weight-for-gestational age: Roles of cord blood vitamin D levels. Sci. Total Environ..

[70] Ene M.C., Tertiu O., Vrancianu O., Chifiriuc M. (2018). Vitamin D status in adult and pediatric Romanian population. Roum. Arch. Microbiol. Immunol..

[71] Niculescu D.A., Capatina C.A.M., Dusceac R., Caragheorgheopol A., Ghemigian A., Poiana C. (2017). Seasonal variation of serum vitamin D levels in Romania. Arch. Osteoporos..

[72] Bucurica S., Prodan I., Pavalean M., Taubner C., Bucurica A., Socol C., Calin R., Ionita-Radu F., Jinga M. (2023). Association of Vitamin D Deficiency and Insufficiency with Pathology in Hospitalized Patients. Diagnostics.

[73] Brîndușe L.A., Eclemea I., Neculau A.E., Cucu M.A. (2024). Vitamin D Status in the Adult Population of Romania—Results of the European Health Examination Survey. Nutrients.

[74] Badiu Tișa I., Cozma-Petruț A., Samașca G., Miere D., Filip L., Banc R., Mîrza O., Iancu M. (2024). Vitamin D Status among 2-18-Year-Old Romanian Pediatric Patients: A Single-Center Study. Nutrients.

[75] Peptine L., Răileanu C.-R., Goroftei L., Verga G.-I., Neagu A., Gurău T., Grigore I., Zaharia A.E., Maftei N.M., Matei M.N. (2023). The prevalence of vitamin D deficiency in a pediatric hospital in Romania. Innov. Rom. Food Biotechnol..

[76] Ministerul Sănătății Ghid Privind Evaluarea şi Terapia Deficitului de Vitamină D la Gravidă, Nou-Născut şi Copil. Monitorul Oficial 24 September 2019; Volume 773. https://legislatie.just.ro/public/DetaliiDocument/218406.

[77] Dragomir R.E., Toader D.O., Gheoca Mutu D.E., Dogaru I.A., Răducu L., Tomescu L.C., Moleriu L.C., Bordianu A., Petre I., Stănculescu R. (2024). Consequences of Maternal Vitamin D Deficiency on Newborn Health. Life.

[78] Dragomir R.E., Gheoca Mutu D.E., Sima R.M., Toader O.D., Stănculescu R.V. (2024). The Impact of Vitamin D Deficiency on Gestational Diabetes Mellitus Risk: A Retrospective Study. Cureus.

[79] Weather & Climate. Average Monthly Sunshine in Sibiu. World Weather & Climate Information; Norwegian Meteorological Institute (MET Norway). https://weather-and-climate.com/average-monthly-hours-Sunshine,Sibiu,Romania.

[80] World Data. Info. Times for Sunrise and Sunset in Romania. https://www.worlddata.info/europe/romania/sunset.php.

[81] Zhang H., Jiang Y., Shi N., Lu Y.Q. (2022). Serum vitamin D levels and acute kidney injury: A systemic review and meta-analysis. Sci. Rep..

[82] Alanazi M., Nabil Aboushady R.M., Kamel A.D. (2022). Association between different levels of maternal vitamin-D status during pregnancy and maternal outcomes. Clin. Nutr. ESPEN.

[83] Bi W.G., Nuyt A.M., Weiler H., Leduc L., Santamaria C., Wei S.Q. (2018). Association Between Vitamin D Supplementation During Pregnancy and Offspring Growth, Morbidity, and Mortality: A Systematic Review and Meta-analysis. JAMA Pediatr..

[84] Al-Beltagi M., Rowiesha M., Elmashad A., Elrifaey S.M., Elhorany H., Koura H.G. (2020). Vitamin D status in preterm neonates and the effects of its supplementation on respiratory distress syndrome. Pediatr. Pulmonol..

[85] Boskabadi H., Abrishami M., Shoeibi N., Sanei Z., Moradi A., Zakerihamidi M. (2022). Comparison of Vitamin D Levels in Premature Infants with and without Retinopathy of Prematurity. Arch. Iran. Med..

[86] Moon R.J., Green H.D., D’Angelo S., Godfrey K.M., Davies J.H., Curtis E.M., Cooper C., Harvey N.C. (2023). The effect of pregnancy vitamin D supplementation on offspring bone mineral density in childhood: A systematic review and meta-analysis. Osteoporos. Int..

[87] Moon R.J., D’Angelo S., Curtis E.M., Ward K.A., Crozier S.R., Schoenmakers I., Javaid M.K., Bishop N.J., Godfrey K.M., Cooper C. (2024). Pregnancy vitamin D supplementation and offspring bone mineral density in childhood follow-up of a randomized controlled trial. Am. J. Clin. Nutr..

[88] Brustad N., Garland J., Thorsen J., Sevelsted A., Krakauer M., Vinding R.K., Stokholm J., Bønnelykke K., Bisgaard H., Chawes B.L. (2020). Effect of High-Dose vs Standard-Dose Vitamin D Supplementation in Pregnancy on Bone Mineralization in Offspring Until Age 6 Years: A Prespecified Secondary Analysis of a Double-Blinded, Randomized Clinical Trial. JAMA Pediatr..

[89] Douros K., Loukou I., Tsabouri S. (2021). More data are needed about vitamin D supplements in pregnancy and infancy including any impact on allergies. Acta Paediatr..

[90] Sammallahti S., Holmlund-Suila E., Zou R., Valkama S., Rosendahl J., Enlund-Cerullo M., Hauta-Alus H., Lahti-Pulkkinen M., El Marroun H., Tiemeier H. (2023). Prenatal maternal and cord blood vitamin D concentrations and negative affectivity in infancy. Eur. Child. Adolesc. Psychiatry.

[91] García-Serna A.M., Morales E. (2020). Neurodevelopmental effects of prenatal vitamin D in humans: Systematic review and meta-analysis. Mol. Psychiatry.

[92] Kim I., Kim S.S., Song J.I., Yoon S.H., Park G.Y., Lee Y.W. (2019). Association between vitamin D level at birth and respiratory morbidities in very-low-birth-weight infants. Korean J. Pediatr..

[93] Kazzi S.N.J., Karnati S., Puthuraya S., Thomas R. (2018). Vitamin D deficiency and respiratory morbidity among African American very low birth weight infants. Early Hum. Dev..

[94] Liu W., Xu P. (2023). The association of serum vitamin D level and neonatal respiratory distress syndrome. Ital. J. Pediatr..

[95] Mohamed Hegazy A., Mohamed Shinkar D., Refaat Mohamed N., Abdalla Gaber H. (2018). Association between serum 25 (OH) vitamin D level at birth and respiratory morbidities among preterm neonates. Matern. Fetal Neonatal Med..

[96] Kim Y.J., Lim G., Lee R., Chung S., Son J.S., Park H.W. (2023). Association between vitamin D level and respiratory distress syndrome: A systematic review and meta-analysis. PLoS ONE.

[97] Dogan P., Ozkan H., Koksal N., Bagci O., Varal I.G. (2020). Vitamin D deficiency and its effect on respiratory distress syndrome in premature infants: Results from a prospective study in a tertiary care centre. Afr. Health Sci..

[98] Zang R., Zhang Y., Zhang H., Zhang X., Lv Y., Li D. (2022). Association Between Vitamin D Level and Neonatal Respiratory Distress Syndrome: A Systematic Review and Meta-Analysis. Front. Pediatr..

[99] Zhou A., Hyppönen E. (2023). Vitamin D deficiency and C-reactive protein: A bidirectional Mendelian randomization study. Int. J. Epidemiol..

[100] Cetinkaya M., Cekmez F., Buyukkale G., Erener-Ercan T., Demir F., Tunc T., Aydın F.N., Aydemir G. (2015). Lower vitamin D levels are associated with increased risk of early-onset neonatal sepsis in term infants. J. Perinatol..

[101] Kamsiah K., Hasibuan B.S., Arto K.S. (2021). The Relationship between Vitamin D Levels and Clinical Outcomes of Neonatal Sepsis in Haji Adam Malik Hospital Medan, Indonesia. Open Access Maced. J. Med. Sci..

[102] Specht I.O., Janbek J., Thorsteinsdottir F., Frederiksen P., Heitmann B.L. (2020). Neonatal vitamin D levels and cognitive ability in young adulthood. Eur. J. Nutr..

[103] Specht I.O., Thorsteinsdottir F., Walker K.C., Olsen J., Heitmann B.L. (2020). Neonatal vitamin D status and risk of childhood epilepsy. Epilepsia.

[104] Yin X., Xu S., Zhang X., Li L., Xi H., Ma L., Sun M., Yang P., Li X., Jiang H. (2024). The association between serum 25-hydroxyvitamin D levels and retinopathy of prematurity in preterm infants. Front. Pediatr..

[105] Matejek T., Zapletalova B., Stepan M., Malakova J., Palicka V. (2023). Dynamics of the vitamin D C3-epimer levels in preterm infants. Clin. Chem. Lab. Med..

[106] Courbebaisse M., Souberbielle J.C., Baptiste A., Taieb J., Tsatsaris V., Guibourdenche J., Senat M.V., Haidar H., Jani J., Guizani M. (2019). Vitamin D status during pregnancy and in cord blood in a large prospective French cohort. Clin. Nutr..

[107] Anderson-Berry A., Thoene M., Wagner J., Lyden E., Jones G., Kaufmann M., Van Ormer M., Hanson C. (2017). Randomized trial of two doses of vitamin D_3_ in preterm infants <32 weeks: Dose impact on achieving desired serum 25(OH)D_3_ in a NICU population. PLoS ONE.

[108] Cooke L.D.F., Tumbarello D.A., Harvey N.C., Sethi J.K., Lewis R.M., Cleal J.K. (2021). Endocytosis in the placenta: An undervalued mediator of placental transfer. Placenta..

[109] Vestergaard A.L., Andersen M.K., Andersen H.H., Bossow K.A., Bor P., Larsen A. (2024). Effects of High-Dose Vitamin D Supplementation on Placental Vitamin D Metabolism and Neonatal Vitamin D Status. Nutrients.

[110] Shadid I.L.C., Guchelaar H.J., Weiss S.T., Mirzakhani H. (2024). Vitamin D beyond the blood: Tissue distribution of vitamin D metabolites after supplementation. Life Sci..

[111] Łukaszkiewicz J. (2016). Vitamin D—Skin synthesis revisited. Nowe spojrzenienasyntezęskórnawitaminy D. Post. N. Med.

[112] Rusińska A., Płudowski P., Walczak M., Borszewska-Kornacka M.K., Bossowski A., Chlebna-Sokół D., Czech-Kowalska J., Dobrzańska A., Franek E., Helwich E. (2018). Vitamin D Supplementation Guidelines for General Population and Groups at Risk of Vitamin D Deficiency in Poland-Recommendations of the Polish Society of Pediatric Endocrinology and Diabetes and the Expert Panel With Participation of National Specialist Consultants and Representatives of Scientific Societies-2018 Update. Front. Endocrinol..

[113] Chirita-Emandi A., Socolov D., Haivas C., Calapiș A., Gheorghiu C., Puiu M. (2015). Vitamin D Status: A Different Story in the Very Young versus the Very Old Romanian Patients. PLoS ONE.

[114] Bhattoa H.P., Nagy E., More C., Kappelmayer J., Balogh A., Kalina E., Antal-Szalmas P. (2013). Prevalence and seasonal variation of hypovitaminosis D and its relationship to bone metabolism in healthy Hungarian men over 50 years of age: The HunMen Study. Osteoporos. Int..

[115] Pludowski P., Grant W.B., Bhattoa H.P., Bayer M., Povoroznyuk V., Rudenka E., Ramanau H., Varbiro S., Rudenka A., Karczmarewicz E. (2014). Vitamin d status in central europe. Int. J. Endocrinol..

[116] Marti D.T., Nesiu A., Balta C., Olariu T.R., Mihu A.G., Hermenean A., Oatis D.A. (2024). Retrospective Analysis of Vitamin D Deficiency in an Adult Population of Arad County, Western Romania (2019–2022). Life.

[117] Rostami M., Tehrani F.R., Simbar M., Bidhendi Yarandi R., Minooee S., Hollis B.W., Hosseinpanah F. (2018). Effectiveness of Prenatal Vitamin D Deficiency Screening and Treatment Program: A Stratified Randomized Field Trial. J. Clin. Endocrinol. Metab..

[118] Taylor S.N. (2024). Vitamin D for very preterm infants-determining the how, when, and why. Pediatr. Res..

[119] Pilz S., Trummer C., Theiler-Schwetz V., Grübler M.R., Verheyen N.D., Odler B., Karras S.N., Zittermann A., März W. (2022). Critical Appraisal of Large Vitamin D Randomized Controlled Trials. Nutrients.

[120] Rostami M., Simbar M., Amiri M., Bidhendi-Yarandi R., Hosseinpanah F., Ramezani Tehrani F. (2021). The optimal cut-off point of vitamin D for pregnancy outcomes using a generalized additive model. Clin. Nutr..

[121] White J.H. (2022). Emerging Roles of Vitamin D-Induced Antimicrobial Peptides in Antiviral Innate Immunity. Nutrients.

[122] Martens P.J., Gysemans C., Verstuyf A., Mathieu A.C. (2020). Vitamin D’s Effect on Immune Function. Nutrients.

[123] Chen Y., Zhang S., Hu L., Dong L., Liu Q., Liu Y., Cheng W., Liu D., Yang G., Li K. (2022). Vitamin D categories and postpartum thyroid function in women with hypothyroidism. Front. Nutr..

[124] Babić Leko M., Jureško I., Rozić I., Pleić N., Gunjača I., Zemunik T. (2023). Vitamin D and the Thyroid: A Critical Review of the Current Evidence. Int. J. Mol. Sci..

[125] Cheng H., Chi P., Zhuang Y., Alifu X., Zhou H., Qiu Y., Huang Y., Zhang L., Ainiwan D., Peng Z. (2023). Association of 25-Hydroxyvitamin D with Preterm Birth and Premature Rupture of Membranes: A Mendelian Randomization Study. Nutrients.

[126] Kiely M., O’Donovan S.M., Kenny L.C., Hourihane J.O., Irvine A.D., Murray D.M. (2017). Vitamin D metabolite concentrations in umbilical cord blood serum and associations with clinical characteristics in a large prospective mother-infant cohort in Ireland. J. Steroid Biochem. Mol. Biol..

[127] Bialy L., Fenton T., Shulhan-Kilroy J., Johnson D.W., McNeil D.A., Hartling L. (2020). Vitamin D supplementation to improve pregnancy and perinatal outcomes: An overview of 42 systematic reviews. BMJ Open.

